# hOA-DN30: a highly effective humanized single-arm MET antibody inducing remission of ‘MET-addicted’ cancers

**DOI:** 10.1186/s13046-022-02320-6

**Published:** 2022-03-29

**Authors:** Ilaria Martinelli, Chiara Modica, Cristina Chiriaco, Cristina Basilico, James M. Hughes, Simona Corso, Silvia Giordano, Paolo M. Comoglio, Elisa Vigna

**Affiliations:** 1 Candiolo Cancer Institute, FPO-IRCCS, Strada Provinciale 142, 10060 Candiolo, TO Italy; 2 Department of Oncology, University of Turin, Turin, Italy

**Keywords:** MET oncogene, Targeted therapy, Antibody, Gastric cancer

## Abstract

**Background:**

The tyrosine kinase receptor encoded by the MET oncogene is a major player in cancer. When MET is responsible for the onset and progression of the transformed phenotype (MET-addicted cancers), an efficient block of its oncogenic activation results in potent tumor growth inhibition.

**Methods:**

Here we describe a molecular engineered MET antibody (hOA-DN30) and validate its pharmacological activity in MET-addicted cancer models in vitro and in vivo. Pharmacokinetics and safety profile in non-human primates have also been assessed.

**Results:**

hOA-DN30 efficiently impaired MET activation and the intracellular signalling cascade by dose and time dependent removal of the receptor from the cell surface (shedding). In vitro, the antibody suppressed cell growth by blocking cell proliferation and by concomitantly inducing cell death in multiple MET-addicted human tumor cell lines. In mice xenografts, hOA-DN30 induced an impressive reduction of tumor masses, with a wide therapeutic window. Moreover, the antibody showed high therapeutic efficacy against patient-derived xenografts generated from MET-addicted gastric tumors, leading to complete tumor regression and long-lasting effects after treatment discontinuation. Finally, hOA-DN30 showed a highly favorable pharmacokinetic profile and substantial tolerability in Cynomolgus monkeys.

**Conclusions:**

hOA-DN30 unique ability to simultaneously erase cell surface MET and release the ‘decoy’ receptor extracellular region results in a paramount MET blocking action. Its remarkable efficacy in a large number of pre-clinical models, as well as its pharmacological features and safety profile in non-human primates, strongly envisage a successful clinical application of this novel single-arm MET therapeutic antibody for the therapy of MET-addicted cancers.

**Supplementary Information:**

The online version contains supplementary material available at 10.1186/s13046-022-02320-6.

## Background

The MET oncogene encodes for a transmembrane tyrosine kinase, the receptor for Hepatocyte Growth Factor (HGF), endowed with pleiotropic functions, including regulation of cell proliferation, motility, invasion, and apoptosis [1–4]. When genetic alterations leading to deregulated MET activation occur (mostly amplification and/or point mutations), MET initiates and maintains cell transformation (MET addiction) [5–8]. MET genetic lesions have been described in different types of solid tumors, with an overall frequency of about 4% (www.cbioportal.org).

MET gene amplification generates a MET receptor constitutively active due to overexpression. Copy number gain can result from focal amplification or polysomy, i.e. in the absence or in the presence of multiple copies of chromosome 7, including the MET gene. The level of MET gene amplification defines tumor cell dependence from the oncogene. Although experimental and clinical data indicate that MET-addiction is reached only in the presence of a high MET gene copy number, a clear cut-off point has still to be defined. MET gene amplification has been found in patients carrying gastric cancers, lung tumors, and in type 1 papillary renal cell carcinomas at a frequency around 10% [9–11], and at a lower frequency (1–5%) in hepatocellular carcinomas, ovarian tumors, melanomas and type 2 papillary renal cell carcinomas [11–14]. In addition to de novo condition, MET gene amplification represents a molecular mechanism responsible for resistance to Epidermal Growth Factor Receptor inhibitors. This has been described in cases of colorectal and lung carcinomas [15–17]. MET gene copy number gain has been also found in cases of resistance to BRAF targeted therapies [18, 19].

Non-synonymous activating point mutations in the MET gene were first described in hereditary and sporadic kidney cancers, involving residues located in the kinase domain [20]. During the last 20 years, an increased number of MET gene mutations have been found in patients carrying different types of cancers (e.g. lung, breast, gastric, hepatocellular, head and neck carcinomas, cancer of unknown primary, see www.cbioportal.org). These amino acid conversions are clustered, in addition to the kinase domain, also in the extracellular portion of the receptor (SEMA domain) or in the juxtamembrane region [21]. While some of these amino acid changes have been functionally validated, the real activating function of others is still debated.

Finally, several genetic alterations have been identified in non-coding regions [22]. These genomic modifications generate alternatively spliced MET mRNAs lacking the exon 14, a region involved in the negative regulation of the receptor enzymatic activity [23]. They have been found in the 3% of Non-Small Cell Lung Cancers (NSCLC) [24] and - at a smaller frequency - in other tumor types.

In light of the role of MET in cancer, several different MET inhibitors have been developed [5, 25, 26]. In the past, we generated and characterized DN30, a murine monoclonal antibody (mAb) displaying both agonist and antagonist properties [27]. In a bivalent antibody format, allowing simultaneous interaction with two distinct antigen molecules, the antibody stabilizes receptor complexes in a way similar to what is achieved by the natural ligand. This leads to partial activation of the MET kinase, which ultimately triggers some - but not all - MET-driven biological responses [27]. On the other hand, DN30 mAb enhances MET proteolytic cleavage, at one single site located within the extracellular domain of the receptor, in a region close to the cell membrane [28]. As a consequence, soluble MET receptors are released in the extracellular space (receptor ‘shedding’) where they can behave as a ‘decoy’, neutralizing HGF and forming inactive heterodimers with bona fide MET receptors which, eventually surviving the cleavage, are still exposed on the cell surface. Inside the cell, the transmembrane MET fragment derived from shedding is further cleaved to detach the kinase-containing cytoplasmatic portion from the membrane. The latter is then rapidly addressed to proteasomal degradation [29]. As a consequence, MET is physically removed from the plasma membrane. This complex cascade of molecular events occurring upon DN30 mAb binding translates into inhibition of MET-driven biological activities. Thus, when DN30 mAb interacts with MET on the cell surface, the net biological response within the cell depends on the balance between two opposing functions: kinase activation and receptor shedding. Disassociation of the two activities has been achieved by conversion of the ancestral bivalent DN30 mAb into a monovalent Fab fragment [30]. This unleashes the therapeutic potential of DN30, leading to a full antagonist molecule. Nevertheless, the short half-life of the Fab fragment limits its clinical development. Thus, to maintain the full antagonist behavior without losing the good pharmacokinetic properties of the immunoglobulins, the conversion from a bivalent to a monovalent antibody was obtained by deleting one of the two antibody arms through a molecular engineering approach. Here we describe the design and validation of the DN30 mAb humanized one-arm form (hOA-DN30), and present data demonstrating its robust anti-proliferative and tumor growth-inhibitory effects in multiple MET-addicted cell lines and xenograft cancer models, as well as its favorable pharmacokinetics and safety profile in non-human primates.

## Methods

More details are provided in Additional File 1.

### hOA-DN30 generation, production, and purification

hOA-DN30 has been provided by Metis Precision Medicine B-Corp (Torino, Italy). Humanization of the DN30 mouse antibody, conversion into the ‘one-arm’ format, production, and purification of hOA-DN30 has been done by Fair Journey Biologics.

### ELISA assays

For affinity determination, the MET extracellular domain fused in frame with a human Fc domain (R&D System) was in the solid phase and pure MvDN30 or hOA-DN30 were in the liquid phase. ELISA signal was quantified by the multi-label reader VICTOR X4 (Perkin Elmer Instrument INC.).

For hOA-DN30 quantification in mouse serum, a goat anti-human IgG antibody (Sigma Aldrich) was in the solid phase. For hOA-DN30 quantification in monkey serum samples, neutravidin-coated plates were used to immobilize biotin-labeled goat anti-human IgG monkey adsorbed antibody (Southern Biotech). In both cases, pure hOA-DN30 and serum samples were in the liquid phase. The concentration of hOA-DN30 in serum was achieved by interpolating the optical density (OD) readings of the samples with the calibration curve fitted by a weighted (1/Y) 4 parameters regression function.

### MET shedding and MET inhibition

For dose-dependent experiments, cells were incubated in serum-free medium for 48 h with the indicated concentration of hOA-DN30. For time-dependent experiments, cells were incubated in serum-free medium in the presence of hOA-DN30 (1 μM) and samples were collected at the indicated time points. Proteins in cell extracts or in cell culture supernatants were resolved by SDS-PAGE and analyzed by Western blotting.

For analysis of MET recovery after shedding, cells were incubated in serum-free medium in the presence of hOA-DN30 (1 μM); after 48 h, cell monolayers were extensively washed and fresh serum-free medium was added. Cell culture supernatants and cell lysates were collected at different time points and analyzed as described above.

For inhibition of MET and downstream signalling pathway activation, cells were treated with the indicated concentrations of hOA-DN30 for 24 h in serum-free medium. Cell monolayers were lysed and analyzed as described above.

### Biological assays

For scatter assay, cells were treated for 24 h with HGF (8 ng/ml, R&D System), hOA-DN30, or DN30 mAb (both 200 nM). Cell scattering was determined by optical microscopy.

For cell growth assay, cells were treated with increasing concentration of hOA-DN30, and viability was evaluated after 72 h by CellTiter-Glo luminescent cell viability assay (Promega Corp.), according to the manufacturer’s instructions. Chemiluminescence was detected with VICTOR X4.

For proliferation assay, cells were treated with hOA-DN30 (1 μM). After 48 h cellular DNA synthesis was determined measuring EdU incorporation using the Click-iT® EdU Alexa Fluor® 647 Flow Cytometry Assay Kit (Thermo Fisher Scientific) according to manufacturer’s instruction.

For cell death assay, cells were treated as described for cell growth assay. Cytotoxicity was evaluated after 72 h by CellTox™ Green Cytotoxicity Assay (Promega Corp., Madison, WI), according to the manufacturer’s instructions. Fluorescence was detected with VICTOR X4 and data normalized over CellTiter-Glo luminescence.

For determination of M192 tumor organoid viability, cells were incubated for 5 h with Alamar blue (Life Technologies). The signal measured in the cell culture supernatants by VICTOR X4 was set as time 0, then freshly prepared medium with 1 or 5 μM hOA-DN30 was added to the culture. Every 3 days, the medium was replaced with a fresh one containing the antibody. After 9 days, a new assessment of the Alamar blue signal was performed. Cell growth was measured calculating the ratio between day 9 and day 0 for each sample.

For determination of Antibody-Dependent Cellular Cytotoxicity (ADCC), cells were treated with increasing concentrations of hOA-DN30, and effector cells were added. After 6 h, ADCC activity has been measured by ADCC Reporter Bioassay (Promega Corp.) according to the manufacturer’s instructions.

### Evaluation of tumor growth inhibition in vivo

All procedures in mice were performed according to protocols approved by the Ethical Committee for animal experimentation of the Candiolo Cancer Institute and by the Italian Ministry of Health.

To generate Cell Derived Xenografts (CDX), cancer cells were injected subcutaneously into the right posterior flank of adult NOD-SCID mice. To generate Patient Derived Xenografts (PDX), tumor materials derived from human gastric tumor specimens expanded for at least 2 generations in mice were implanted in a subcutaneous pocket generated in the flank of NOD-SCID mice as described in [31]. When tumors were established, animals were divided into experimental arms homogeneous for tumor size and randomly assigned to the different treatments. hOA-DN30 was administered by intravenous injection. Tumor size was evaluated periodically with a caliper. Tumor volume was calculated as described [30].

### Pharmacokinetics, pharmacodynamics, and toxicological studies

For pharmacokinetic (PK) evaluation in mice, adult male NOD/SCID mice bearing or not EBC-1 tumors were used. Animals were treated with 30 mg/kg of hOA-DN30 in a single intravenous administration. Animals were bled at different time points. hOA-DN30 levels in mouse serum were determined by ELISA assay (see above) by Accelera Srl. (Nerviano, Italy). PK analysis was performed according to standard non-compartmental and compartmental approach using Phoenix-WinNonlin package (v. 6.3, Pharsight Inc., Certara Company) by Accelera Srl.

For PK evaluation in Cynomolgus monkeys, the experiments have been performed by Accelera Srl. Three adult male monkeys were administered with hOA-DN30 as a single intravenous bolus at the dose of 11 mg/kg and animals were bled at different time points. hOA-DN30 concentrations in serum were determined by ELISA assay (see above). PK analysis was performed according to the standard non-compartmental approach as described above.

For pharmacodynamics (PD), the analysis was performed by Accelera Srl, applying an E_max_ (maximum kill rate) PK/PD model [32] to tumor volumes measured in mice treated with the indicated doses of hOA-DN30. The threshold systemic (plasma) concentration for tumour stabilization (C_τ_) was calculated from the equilibrium status of the first differential equation of the model.

Determination of hOA-DN30 tolerability was performed by Accelera Srl. The antibody was administered intravenously according to ascending doses (30, 90, and 180 mg/kg) one week apart, or at the dose of 180 mg/mg at weekly intervals for two times to two adult Cynomolgus monkeys (one male, one female). Animal’s healthy status, clinical signs observation, body weights, and food consumption were evaluated periodically. Standard hematological and serum chemical parameters were determined. At necropsy, body weight measurement and macroscopic examinations were done.

All the studies performed by Accelera Srl. have been sponsored by Metis Precision Medicine B-Corp.

### Statistical analysis

Averages, standard deviations, and *P* values obtained by Student’s t-Test were calculated using Microsoft Office Excel 2010 software (Microsoft Corporation). To calculate Kd and B_max_, data from ELISA assay were analyzed and fitted according to nonlinear regression, one-site binding hyperbola curve, using GraphPad Prism software (GraphPad Software). To calculate IC_50_, data from growth assays were analyzed and fitted according to a nonlinear regression, sigmoidal dose-response curve, using GraphPad Prism software. *P* values obtained by One-way or Two-way Anova were calculated using GraphPad Prism software.

## Results

### Generation and characterization of hOA-DN30, a humanized ‘one-arm’ form derived from the DN30 mAb

**Fig. 1:**
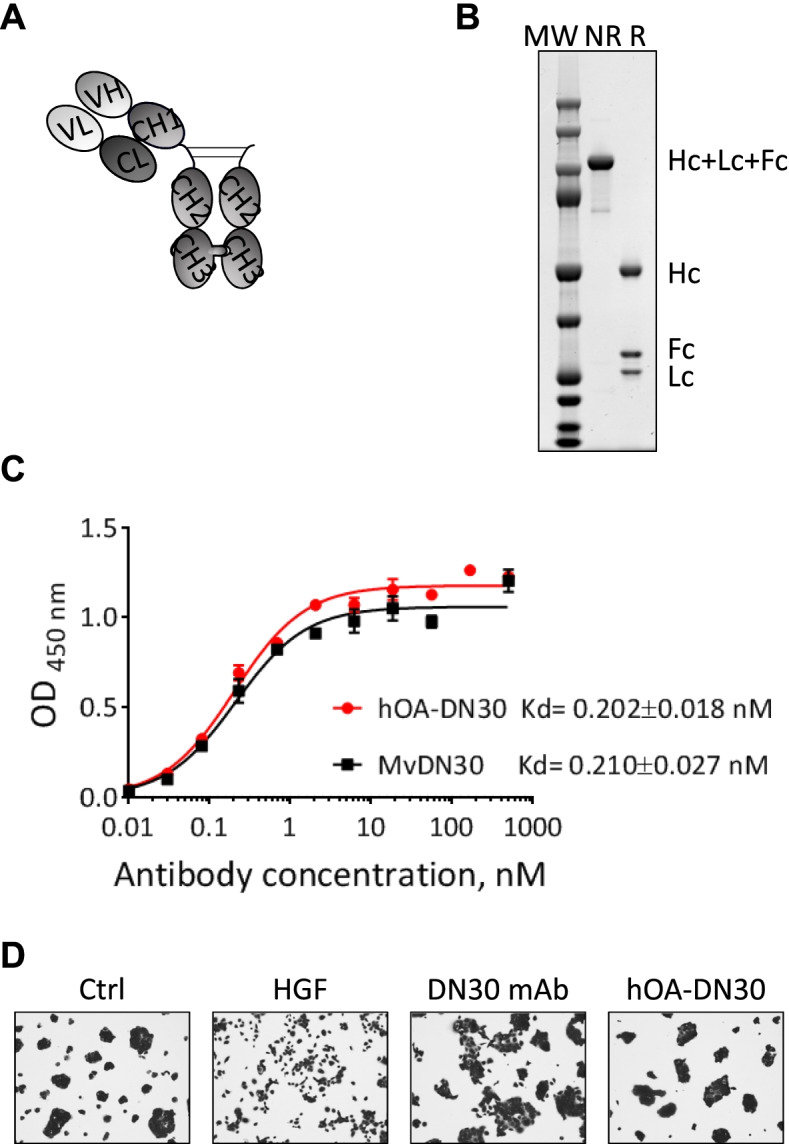
Analysis of hOA-DN30 structure and function. **a** Schematic representation of hOA-DN30. VH: humanized variable domain of the heavy chain. CH1, CH2, CH3: constant domains derived from human IgG1 heavy chain. VL: humanized variable domain of the light chain. CL: constant domain derived from human Igk light chain. **b** SDS-PAGE in polyacrylamide gel under non-reducing (NR) and reducing (R) conditions, followed by staining with GelCode Blue Stain reagent. MW: Molecular Weight; Hc: Heavy chain; Lc Light chain; Fc: fragment crystallizable region. **c** ELISA binding analysis of hOA-DN30 and MvDN30 (liquid phase) to a Met-Fc chimera (solid phase). OD, optical density at 450 nm. Dissociation constant values (Kd) ± SD are reported in the graph. Each point is the mean of triplicate values; bars represent SD. **d** HPAF-II cells untreated (Ctrl) or treated with HGF (8 ng/ml), or bivalent murine DN30 mAb (200 nM), or hOA-DN30 (200 nM) for 18 h. Pictures are representative of the cell phenotype: control cells grew in compact islands, while HGF-treated cells appeared as single cells separated from each other (scattered). Some scattered islands were found in the chimeric DN30 mAb treated cells. Stimulation with hOA-DN30 did not induce cell motility, appearing indistinguishable from controls. Data reported in the figure are representative of at least two experiments done

The hOA-DN30 molecule was generated from the sequence of the DN30 mAb [33], modified to obtain a humanized antibody characterized by: (i) a monovalent structure, achieved by assembling three different polypeptides, i.e. one antibody light chain, one full-length IgG1 heavy chain, and one Fc region (Fig. 1A); (ii) introduction of specific amino acid modifications in the CH3 domains of the heavy chain and of the Fc domain, to produce the ‘*knob into hole’* structure [34]; (iii) a high degree of homology/identity with human germline antibody sequences (91.75% and 93.4%, respectively). The recombinant purified product (Fig. 1B) has been tested for MET binding by ELISA assay. hOA-DN30 interacted with MET with high affinity; the calculated constant of dissociation (Kd) was similar to its parental reference molecule, the chimeric Fab fragment (MvDN30) [35] (Fig. 1C). No residual MET agonistic activity was observed by assessing hOA-DN30 ligand mimetic properties in scatter assay, a highly sensitive procedure to evaluate the induction of MET-mediated responses in the cells (Fig. 1D).

### hOA-DN30 potently induces MET ‘shedding’ and strongly inhibits MET activation

**Fig. 2:**
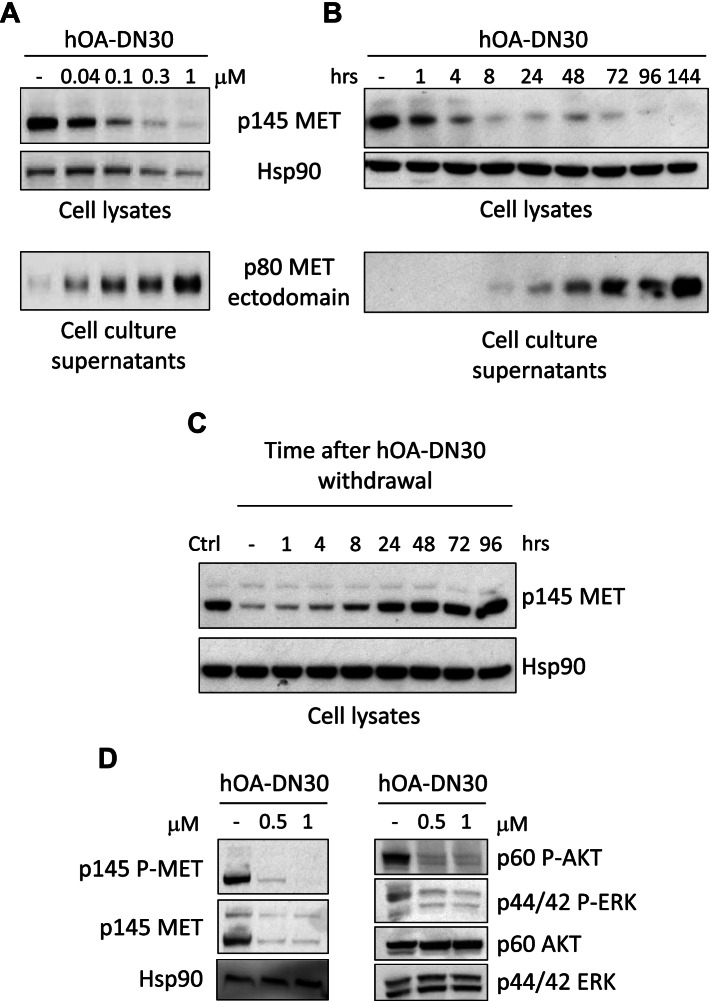
Analysis of hOA-DN30 MET inhibitory properties. **a** A549 cells treated with increasing concentrations of hOA-DN30 for 72 h. **b** A549 cells treated with 1 μM hOA-DN30 for the indicated times. **c** A549 cells treated with 1 μM hOA-DN30 for 48 h and then left untreated for the indicated times. **d** GTL-16 cells treated with increasing concentrations of hOA-DN30 for 24 h. MET, AKT, ERK protein levels and activation were determined by Western blot analysis of cell lysates. MET ectodomain levels were determined by Western blot analysis of cell culture supernatants. To normalize protein loading, filters were re-probed with anti-Hsp90. p145 MET: MET receptor β chain; p145 P-MET: phosphorylated MET receptor β chain; p80 MET: MET-ECD; p60 AKT: AKT; p60 P-AKT: phosphorylated AKT; p44/42 ERK: ERK; p44/42 P-ERK: phosphorylated ERK; Hsp90: Heat shock protein. Data reported in the figure are representative of at least two experiments

The ability of hOA-DN30 to promote MET receptor shedding and downregulation was evaluated in A549 NSCLC cells. hOA-DN30 efficiently induced MET shedding, resulting in a dose-dependent release of soluble MET ectodomains (ECDs) in the extracellular space, and in concomitant progressive elimination of MET receptor from the cells (Fig. 2A). At the highest dose tested (1 μM), MET depletion was detected starting from 1 h of antibody treatment, and after 4 days MET was erased from the cell, while accumulation of MET-ECDs in the extracellular environment was detectable after 8 h (Fig. 2B). After hOA-DN30 withdrawal, MET expression was recovered within 24 h (Fig. 2C). Kinetics of hOA-DN30-induced MET shedding and of MET expression recovery after antibody treatment were evaluated also in MET-amplified EBC-1 human NSCLC cells (Suppl. Fig. 1A and B). As a consequence of receptor over-expression due to gene amplification, human gastric carcinoma GTL-16 cells feature a constitutively active MET, highly phosphorylated in the absence of HGF [36]. Upon hOA-DN30 treatment, MET phosphorylation dropped-off according to the antibody given dose (Fig. 2D, left panels). In parallel, also the signalling pathway downstream MET was impaired, as assessed by phosphorylation levels of AKT and ERK, the two major nodes of the MET-triggered intracellular signalling cascade (Fig. 2D, right panels). Inhibition of MET and intracellular transducers activation was assessed also in EBC-1 cells (Suppl. Fig. 1C).

### hOA-DN30 powerfully inhibits MET-addicted cell growth, blocking proliferation and inducing cell death

**Fig. 3:**
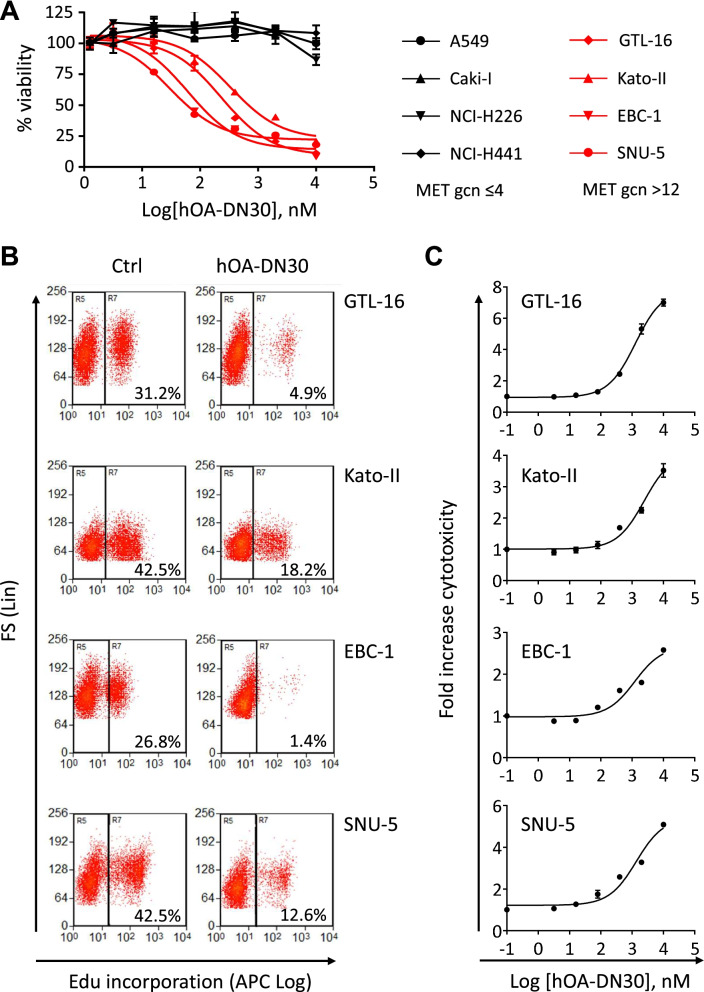
Analysis of hOA-DN30 cancer cell growth inhibition in vitro. **a** Analysis of cell viability by quantitation of metabolically active cells in a panel of cancer cell lines treated with increasing concentrations of hOA-DN30 for 72 h. Black: cancer cells carrying diploid or low amplified MET gene; Red: cancer cells carrying high MET gene amplification. Gcn: gene copy number. For each cell line, viable cells are reported as the percentage of untreated cells. Each point is the mean of triplicate values; bars represent SD. **b** Analysis of cell proliferation by EdU incorporation. Cytofluorimeter analysis of the indicated MET-amplified cells treated with 1 μM hOA-DN30 for 48 h. Ctrl: untreated cells. The percentage of EdU-positive cells is indicated in the plots. FS: Forward Scatter. **c** Analysis of cell death by incorporation of CellTox Green Dye. MET-amplified cells treated with increasing concentrations of hOA-DN30 for 72 h. Graphs report the fold increase of cytotoxicity versus untreated cells. Each point is the mean of triplicate values; bars represent SD

A panel of exponentially growing human carcinoma cells, characterized by a different MET gene copy number (Suppl. Table 1), were treated with increasing concentrations of hOA-DN30. The antibody inhibited the growth of all cancer cells carrying high MET gene amplification (Fig. 3A). The calculated IC_50_ values were in the nanomolar range (Suppl. Fig. 2). Conversely, hOA-DN30 was not effective in cells carrying diploid or low amplified wild-type MET (Fig. 3A). To further analyze the mechanisms underlining the growth inhibition exerted by hOA-DN30, cell proliferation and cell death upon antibody treatment were evaluated in the highly MET-amplified cell lines. hOA-DN30 greatly affected cell proliferation, measured by the incorporation of EdU during the S phase of the cell cycle (Fig. 3B) and induced cell death (Fig. 3C). Moreover, dose-dependent inhibition of cell growth by hOA-DN30 was established in organoids derived from human tumor tissue expanded in mice after subcutaneous implantation of bioptic material derived from a colon cancer patient characterized by high MET gene amplification (PDX M192) [16], (Suppl. Fig. 3). To investigate if the antitumor activity of hOA-DN30 could be further enhanced by triggering Antibody-Dependent Cellular Cytotoxicity (ADCC), we performed an in vitro test incubating the highly MET-amplified EBC-1 and GTL-16 cells with the antibody, in the presence of effector cells. Data reported in Suppl. Fig. 4 show that hOA-DN30 did not exert this function. Finally, a preliminary study was conducted to assess the activity of hOA-DN30 in comparison to ABT-700, a MET antibody tested in the clinic (NCT01472016). This antibody interacts with the IPT-1 domain of MET and displays a mechanism of action different from hOA-DN30, as it inhibits both HGF- dependent and independent MET activation by impairing HGF binding and counteracting receptor dimerization [37, 38]. hOA-DN30 induced a higher maximal inhibition of MET-addicted EBC-1 cell growth compared to ABT-700 (Suppl. Fig. 5).

### hOA-DN30 impairs growth of MET-addicted tumors in vivo

**Fig. 4:**
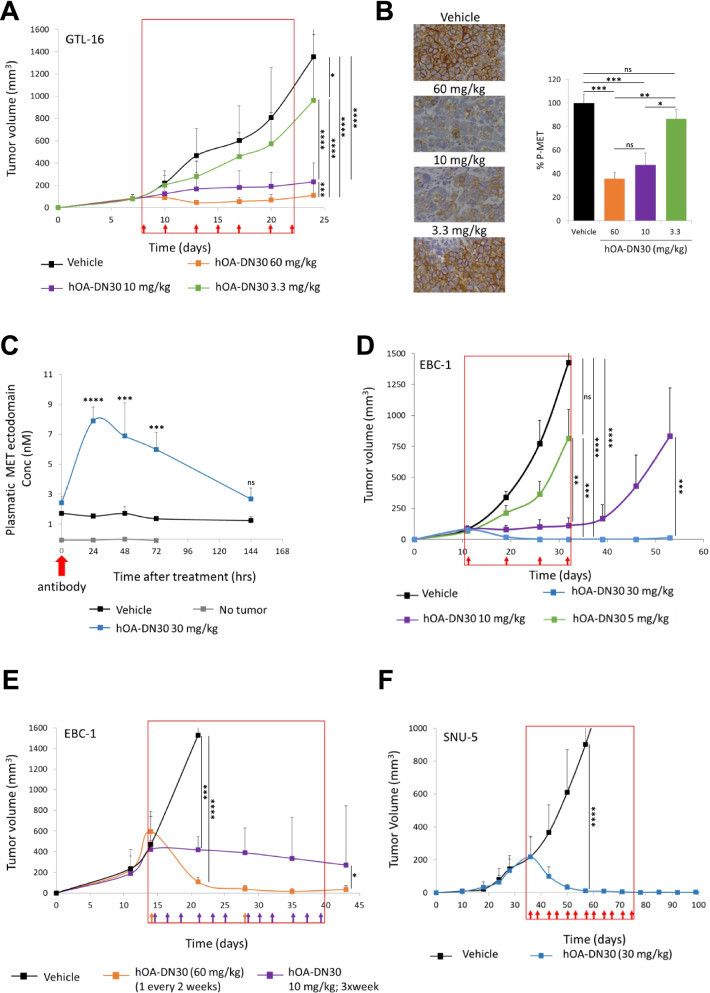
Analysis of hOA-DN30 tumor inhibition in vivo. **a** Analysis of tumor growth in NOD-SCID mice subcutaneously injected with GTL-16 MET-addicted gastric carcinoma cells treated with increasing concentrations (3.3, 10, 60 mg/kg) of hOA-DN30 3xweek. **b** Analysis of MET phosphorylation in tumors collected at the end of the experiment shown in panel a. Left, representative images; right, quantification of the staining represented as the percentage with respect to untreated tumors. Bars represent SEM. **c** Quantification of circulating human MET-ECDs in tumor-bearing NOD-SCID mice treated with hOA-DN30 (30 mg/kg). **d** Analysis of tumor growth in NOD-SCID mice subcutaneously injected with EBC-1 MET-addicted lung carcinoma cells treated with increasing concentrations (5, 10, 30 mg/kg) of hOA-DN30 1xweek. **e** Analysis of tumor growth in NOD-SCID mice subcutaneously injected with EBC-1 MET-addicted lung carcinoma cells treated with the same total amount of hOA-DN30 administered with different schedules (60 mg/kg 1x2weeks or 10 mg/kg 3xweek). **f** Analysis of tumor growth in NOD-SCID mice subcutaneously injected with SNU-5 MET-addicted gastric cancer cells treated with 30 mg/kg of hOA-DN30 2xweek. Red boxes: period of hOA-DN30 administration; arrows: antibody deliveries; bars represent SD. Statistical significance evaluated by One-way Anova analysis (panel **b**) or Two-way Anova analysis (panels **a**, **c**-**f**) is reported; ****, *p *<0.0001; ***, *p*<0.001; **, *p*<0.01; *, *p*<0.05

The ability of hOA-DN30 to inhibit tumor growth in vivo was tested on different MET-addicted Cell line Derived Xenograft (CDX) models, characterized by high levels of MET gene amplification (Suppl. Table 1). hOA-DN30 showed a therapeutic effect, inhibiting GTL-16 tumor growth with a dose-response behavior (29%, 83%, and 92% inhibition at 3.3, 10, and 60 mg/kg three times a week, respectively; Fig. 4A). Inhibition of cancer cell proliferation has been confirmed by Ki67 staining on the tumor masses upon sacrifice (Suppl. Fig. 6A). Dose-dependent down-modulation of the target in vivo was assessed by evaluation of MET phosphorylation (Fig. 4B) and MET protein levels (Suppl. Fig. 6B) within the tumors at sacrifice. Moreover, we proved the occurrence of MET shedding in vivo by measuring the amount of circulating human MET-ECD at different time points after hOA-DN30 treatment (Fig. 4C). Dose-dependent efficacy was also assessed in the EBC-1 CDX model. The hOA-DN30 dose of 30 mg/kg once a week was sufficient to induce complete tumor remission in all treated mice, and the effect persisted for a long time even after treatment discontinuation (Fig. 4D). Furthermore, EBC-1 tumors were strongly responsive to hOA-DN30 treatment, independently from the size of the tumor masses at first treatment (Suppl. Fig. 7A). To explore the impact of the treatment schedule on the therapeutic response to hOA-DN30, an equal total amount of antibody (2400 μg/mouse), fractionated in 2 or 12 administrations, was delivered to mice carrying EBC-1 tumors. Data reported in Fig. 4E show that few administrations at higher doses induced a better therapeutic response compared with frequent administrations at lower doses. On a third CDX model, SNU-5, hOA-DN30 was extremely effective, inducing complete tumor remission; moreover, upon antibody withdrawal, no tumor recurrence was observed (Fig. 4F). Anti-tumor efficacy was also observed against Hs746T, a gastric cancer CDX model featuring both MET-amplification and exon 14 skipping mutations (Suppl Fig. 7B). It is well known that the tumor microenvironment can favor tumor growth and can influence the response to anti-cancer molecules [39]. We previously reported that modifications in cancer cell metabolism instruct cancer-associated fibroblasts to increase HGF production, sustaining an adaptive resistance response [40]. To test if hOA-DN30 therapeutic activity could be impaired in the presence of HGF overexpression, tumors obtained by injection of EBC-1 cells featuring the above described adaptive resistance (EBC-1_Res) were treated with the antibody. Data reported in Suppl. Fig. 8 demonstrate that hOA-DN30 is active in breaking non-cell-autonomous resistance.

### hOA-DN30 induces complete and long-lasting remission of MET- highly amplified patient-derived gastric cancer xenografts

**Fig. 5:**
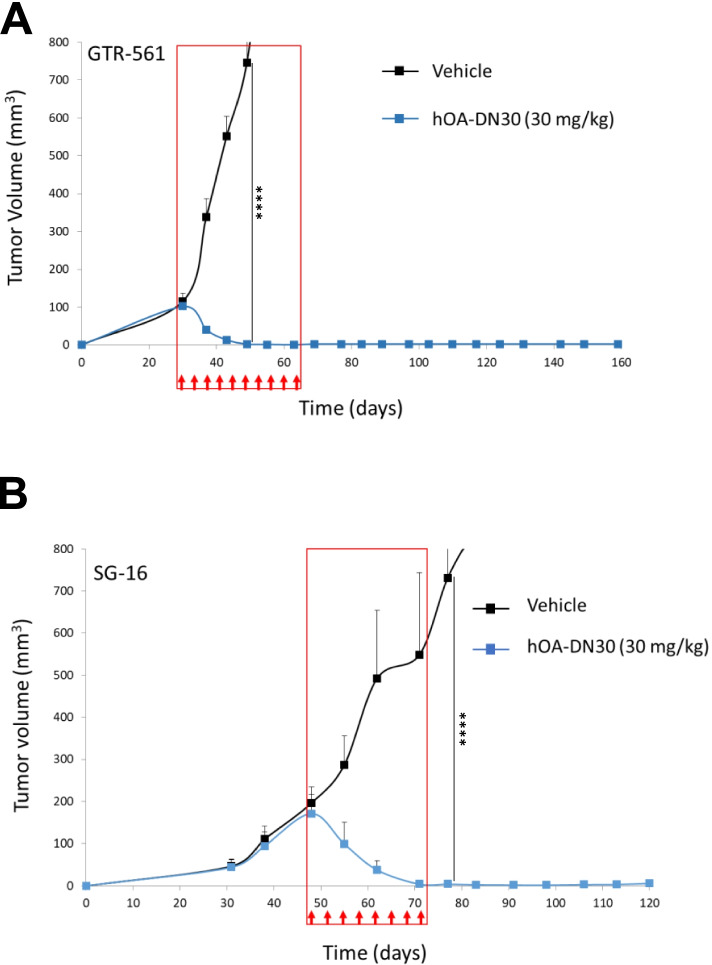
Analysis of patient-derived gastric tumor growth in experimental animals treated with hOA-DN30. **a** Analysis of tumor growth in NOD-SCID mice subcutaneously implanted with GTR-561 gastric PDX treated with 30 mg/kg of hOA-DN30 2xweek. **b** Analysis of tumor growth in NOD-SCID mice subcutaneously implanted with SG-16 gastric PDX treated with hOA-DN30 (30 mg/kg, 2xweek). Red boxes: period of hOA-DN30 administration; red arrows: antibody deliveries; bars represent SD. Statistical significance evaluated by Two-way Anova analysis is reported; ****, *p*<0.0001

Finally, hOA-DN30 activity was evaluated against MET-amplified Patient Derived Xenografts (PDXs) of gastric cancer origin. MET gene copy number for each PDX is reported in Suppl. Table 1. In the GTR-561 model, characterized by a high MET gene copy number, tumors masses shrank very fast and completely disappeared. Upon suspension of the treatment, the antibody effect was extremely long-lasting; mice survived tumor-free for more than 90 days (Fig. 5A). A similar complete and durable tumor remission was observed also in SG-16, a second PDX model featuring high MET gene amplification (Fig. 5B). On the contrary, and in line with what was observed in vitro, the growth of PDXs characterized by a low grade of MET gene amplification was not impaired by hOA-DN30 treatment (Suppl. Fig. 9).

### hOA-DN30 shows a favorable pharmacokinetic profile and no toxic effects in vivo

**Fig. 6:**
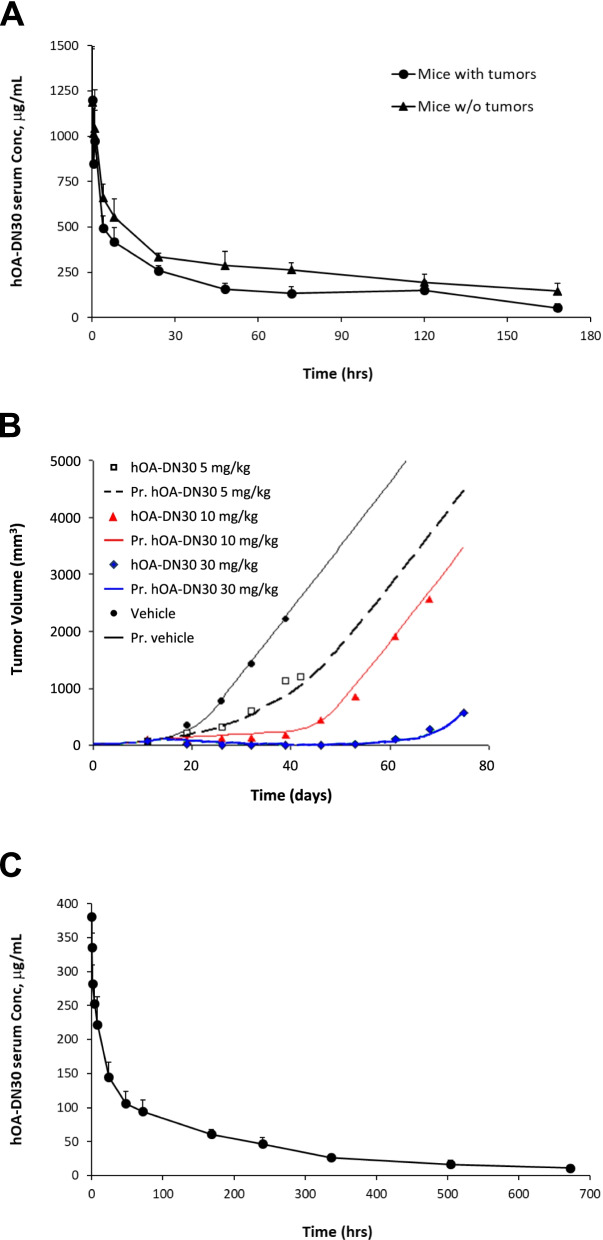
Pharmacokinetics/pharmacodynamics of hOA-DN30. **a** Serum concentrations measured at different time points after single intravenous administration of hOA-DN30 (30 mg/kg) to NOD-SCID mice bearing or not EBC-1 tumors. Each point is the mean of quadruplicate values; bars represent SD. **b** Observed tumor volumes and predicted (Pr.) tumor growth curves after intravenous administrations of hOA-DN30 (5, 10, and 30 mg/kg 1xweek) to NOD-SCID mice bearing subcutaneous EBC-1 tumors. **c** Serum concentrations measured at different time points after single intravenous administration of hOA-DN30 (11 mg/kg) to Cynomolgus monkeys. Each point is the mean of triplicate values; bars represent SD

hOA-DN30 underwent preclinical studies to evaluate its pharmacokinetic (PK) properties in mice and in non-human primates. PK in mice was performed by administering a single dose (30 mg/kg) of the antibody by intravenous injection to immune-deficient mice bearing or not tumors. hOA-DN30 levels in serum samples are shown in (Fig. 6A). A PK profile was obtained by applying both non-compartmental and compartmental analysis. Results from the two different methods were in agreement (Suppl. Tables 2 and 3), showing low clearance (0.42 mL/h/kg), low volume of distribution (66–70 mL/kg), and prolonged half-life (approximately 5 days). Comparing data acquired from healthy mice with those obtained from tumor-bearing animals, the first decline phase was comparable between the two groups, as well as the volume of distribution, while differences were scored in terms of clearance (higher in mice with tumors), terminal half-life and mean residence time (both shorter in mice with tumors).

Data from the in vivo study performed on EBC-1 tumors treated with different doses of the antibody (see above Fig. 4D), were compared to the theoretical results obtained applying a PK/PD model developed by using an E_max_ (maximum kill rate) model [32]. The analysis showed that the model correctly describes tumor volume variations, in all the conditions tested (Fig. 6B). The mean values of the PK parameters obtained in tumor-bearing mice treated with 30 mg/kg of hOA-DN30 were used to simulate inside the PK/PD model the concentration-time profile of the antibody at the ascending doses of the compound. From this analysis, the in vivo serum concentration of hOA-DN30 maintaining tumor growth stabilization (Cτ) was estimated to be 72.9 μg/mL. Notably, administering the dose of 30 mg/kg, serum levels of the compound were above the Cτ threshold throughout the whole treatment (Suppl. Fig. 10).

The PK profile was also analyzed in non-human primates, by delivering hOA-DN30 as a single intravenous bolus at the dose of 11 mg/kg to male Cynomolgus monkeys. The serum concentration of the antibody declined according to a poly-exponential curve (Fig. 6C). The PK profile of the compound was characterized by low serum clearance (0.32 mL/h/kg), low volume of distribution (80 mL/kg), and long terminal half-life (8 days), in good agreement with data obtained in the mouse. PK parameters are reported in Suppl. Table 4.

To define the preclinical model/s suitable for studying antibody safety, we assessed hOA-DN30 binding properties versus MET derived from different species. hOA-DN30 cross-reacted with the MET receptor of human, rat, dog, and monkey origin, while the interaction with mouse MET was very weak (Suppl. Fig. 11A and B). Thus, the Cynomolgus monkey model was selected to perform a dose-escalation study. hOA-DN30, administered according to ascending doses (30, 90, and 180 mg/kg) once a week, was well tolerated. There were no significant toxicological observations or changes in hematology or blood chemistry analysis during the period of antibody administration. Moreover, no late-onset toxicity was observed during a two weeks dosing-free recovery period. Based on these data, we performed a second study in which the highest dose (180 mg/kg) was administered twice, at a weekly interval. Also during this study, no relevant change in body weight (Suppl. Fig. 12), food intake, clinical pathology, and gross examination were observed. Thus, the dose of 180 mg/kg was established as the no observed adverse effect level (NOAEL).

## Discussion

From the outset of precision medicine, MET has been considered an attractive target for cancer therapy [41]. As a consequence, a variety of molecules against either the MET receptor or its specific ligand, HGF, have been explored during the last 15 years [5, 26]. While most of them stopped at the preclinical phase, a reasonable number of small molecules and antibodies entered clinical trials, with different grades of success.

Crizotinib, a multi-targeting MET Tyrosine Kinase Inhibitor (TKI) [42], gave positive clinical outcomes in case reports of patients sharing the presence of MET-gene amplification in the tumor [18, 43–45]. Crizotinib activity against MET high-amplified tumors has been also confirmed in a retrospective study in NSCLC patients re-classified according to MET/CEP7 ratio [46]. Recently, positive results have been obtained in Phase II clinical trials conducted in NSCLC patients with Capmatinib and Tepotinib, two selective MET inhibitors [47, 48]. These data show that a fraction of patients was responsive to the treatment, and this portion further increases in the case of naïve patients [49, 50]. Nevertheless, the use of TKIs intrinsically brings some drawbacks. Tumors treated with small molecules inevitably and rapidly become resistant to the therapy [51–53]. Moreover, small molecule discontinuation can induce hyper-activation of the kinase, leading to a disease flare [54, 55]. Lastly, TKI toxicity, especially when combination regimens are required, is common [56]. Thus, MET inhibitory antibodies have still the potential to provide a beneficial efficacy and safety outcome in patients.

The first MET antibody tested in the clinic was Onartuzumab, a potent HGF competitor antibody efficiently blocking HGF-dependent MET activation [57]. So far, these studies, as well as those conducted with anti-HGF antibodies, resulted in poor or null benefits [58–61]. These failures rely mainly on the mechanism of action of the above molecules. Indeed, MET-addicted tumors, which are those potentially eligible for a MET-targeted therapy, are characterized by the presence of activating genetic lesions (gene amplification or point mutations), making receptor activation independent from its ligand. In this condition, MET antibodies exclusively competing for ligand binding, as well as HGF-targeting antibodies, are intrinsically ineffective.

hOA-DN30 displays a novel mechanism of action completely different from the simple inhibition of ligand/receptor interaction; its binding epitope - the IPT4 region of MET [35] - does not overlap with HGF binding sites [27]. hOA-DN30 activity relies on the peculiar property, not shown for any other anti-MET drug, of enhancing MET shedding [28]. Shedding, a physiologic cellular mechanism of protein degradation, is exploited by the cells to tightly regulate receptor signalling [62]. MET shedding is predominantly operated by a surface metalloprotease - ADAM-10 - that cleaves the extracellular domain of MET, recognizing a specific sequence immediately upstream of the trans-membrane moiety [63]. Upon shedding, a cascade of events takes place; the membrane-linked MET C-terminal fragment becomes the substrate of a transmembrane protease, γ-secretase, which detaches the intracellular kinase-domain from the cell membrane. This MET portion is rapidly shuttled towards the proteasome [29]. Moreover, an alternative lysosomal-dependent second route of MET degradation is also activated [64]. As a consequence of the above proteolytic events, the net number of MET receptors exposed at the cell surface is strongly reduced and, concomitantly, MET-ECDs are released in the surrounding space. As they are fully competent for ligand binding, MET-ECDs can sequester HGF in the extracellular environment. In addition, MET-ECDs can dimerize with ‘cleavage-survived’ MET receptors still exposed in the plasma membrane, impairing kinase trans-activation [65]. In summary, the binding of hOA-DN30 to MET triggers a complex response acting on the HGF/MET axis at different levels. Thus, unlike other modes of action, the effects of hOA-DN30 on the HGF/MET axis may be deeper and longer sustained.

Currently, a new generation of MET antibodies featuring mechanisms of action different from Onartuzumab is under clinical evaluation.

Teliso-V/ABBV-399 is an antibody-drug conjugate that displays inhibitory effects against MET-overexpressing cells [66] mainly - if not only - thanks to the activity of the anti-mitotic drug. Thus, the antibody represents a tool to deliver a cytotoxin to tumor cells characterized by high MET expression, regardless of their reliance on MET signalling. In our study, we demonstrate that the activity of hOA-DN30 is highly restricted to MET-addicted cancers. Although this condition is expected in a reduced proportion of tumors (see www.cbioportal.org), this should be considered a *plus* and not a limitation, as it assures a personalized response when a correct genetically-based patient selection is applied. On the contrary, patient selection based uniquely on the level of MET expression does not seem reliable [67].

Other antibodies tested so far in humans - SAIT-301, Emibetuzumab, ARGX-111, and a mixture of two antibodies, Sym-015 - are able to block HGF binding and concomitantly induce MET internalization [68–71]. These bivalent antibodies share a common feature: they interact with MET in a region overlapping the binding site for the HGF beta chain. This ligand/receptor interaction, although occurring with low affinity, is crucial as it drives the biological responses [72]. Antibodies binding at this site productively interfere with HGF activity and, thanks to the ability to downregulate MET, also impair ligand-independent kinase activation. Nevertheless, they still retain some agonistic properties - measured as receptor phosphorylation and/or signalling pathway activation – due to induction, even transient, of receptor dimerization [69–71, 73]. Such residual ligand mimetic activity can be considered a potential issue during the clinical application, as it might produce unwanted side effects. Therefore, only monovalent antibodies, like hOA-DN30, behaving as pure antagonists, guarantee a univocal outcome.

In addition to ligand competition and receptor downregulation properties, ARGX-111 and Sym-015 also unleash the immune response of the host against the tumor by triggering ADCC [68, 74]. We proved that ADCC does not contribute to the anti-tumor effect of hOA-DN30. This inability is probably related to the mechanism of action of hOA-DN30. By inducing MET shedding, the antibody does not accumulate at the cell membrane in complex with its target, and thus it cannot mediate the interaction between killer cells and tumor cells. Importantly, the absence of ADCC activity represents a safety benefit, minimizing the risk of adverse immune effects against normal tissues.

Besides antibodies with a canonical structure, a biparatopic antibody, REGN-5093, is currently under clinical testing. This molecule not only blocks HGF/MET binding and induces receptor internalization, but also modulates receptor trafficking [75]. Due to the biparatopic structure, the interaction between REGN-5093 and MET gives rise to large complexes that, not entering in the recycling tubules, are mainly included in the multivesicular endosomes and then degraded in the lysosomes. Nevertheless, receptor recycling is only partially affected; a quote of receptors - estimated as 25% of the total internalized molecules - still goes back to the membrane. This leakiness of the mechanism of action is reflected in the antibody efficacy; tumor regression is very slow and requires prolonged treatment [75]. On the contrary, the therapeutic response observed upon hOA-DN30, relying on an enzymatic response, is quite fast and long-lasting.

In summary, hOA-DN30 possesses unique features when compared to anti-MET antibodies in clinical development. The molecule being monovalent is a pure antagonist. hOA-DN30 mechanism of action (i.e. MET shedding) is based on an initial proteolytic event, the MET cleavage, which prompts intracellular receptor destruction not dependent on receptor trafficking. This activity should be considered more robust and definitive compared with the receptor internalization induced by other MET inhibitory antibodies. In the case of internalization, the receptor can be recycled from the endosomes, while, in the case of shedding, the recovery of MET on the cell surface relies exclusively on new receptor synthesis. Thus, MET turn-over is rapid in the case of internalizing antibodies and, conversely, delayed in the case of hOA-DN30, assuring a longer-lasting effect. MET shedding not only removes MET from the cell surface but also intrinsically triggers other mechanisms able to block the activation of MET receptors that survived the cleavage. This occurs by releasing MET-ECDs in the microenvironment. The ‘decoy’ effect of MET-ECDs is multi-faceted, being effective on the side of ligand-independent MET-activation, by forming inactive receptor heterodimers, as well as on the side of ligand-dependent MET activation, sponging HGF. Notably, MET-ECDs represent the best option to neutralize the ligand, as they incorporate all the binding features included in the natural receptor.

From a pharmacological point of view, hOA-DN30 displays a considerably improved profile compared to its parental molecules (DN30mAb and MvDN30) [76], not dissimilar from those reported for other therapeutic antibodies featuring the typical bivalent structure [77]. Moreover, the one-arm structure of the molecule should not limit the duration of the therapeutic response, as no antibodies against the knob and hole regions have been detected, neither in monkeys nor in humans [57]. The half-life and clearance values of hOA-DN30 indicate a low rate of antibody elimination from the systemic circulation. The volume of distribution measured in the monkey denotes a limited distribution in normal tissues, while the higher clearance rate in tumor-bearing mice compared to the healthy ones alludes to a promising accumulation of the antibody at the tumor site. Dose response and dose regimen experiments in mice suggest that hOA-DN30 can potentially operate with a wide therapeutic window.

From a toxicological point of view, the absence of adverse effects in the monkey at the highest dose tested (180 mg/kg) is extremely encouraging. The good tolerability of hOA-DN30 is in line with what was reported for other MET therapeutic antibodies in non-human primate preclinical models [69, 71] and in clinical trials [78–80] (Sym 015: Camidge R JCO 38 issue 15 suppl. ASCO 2020 abstract 9510). The only observed side effects were, in general, similar to those reported in patients treated with antibodies directed against other receptor tyrosine kinases, thus suggesting that MET targeting does not elicit major complications.

## Conclusions

Ultimately, hOA-DN30 possesses features resulting in a paramount MET blocking action. The intense and robust therapeutic response exerted by hOA-DN30 in a large number of preclinical models, as well as its pharmacological features and safety profile in non-human primates, strongly envisage a successful clinical application of this novel single-arm MET therapeutic antibody.

## Supplementary Information

**Additional file 1.**

**Additional file 2.**

**Additional file 3.**

## Data Availability

All data related to this study are included in this paper and its supplementary information files. No data sets were generated or analyzed during the current study. Experimental details are available from the corresponding author on reasonable request. hOA-DN30 must be requested to Metis Precision Medicine B-Corp (Torino, Italy) m.brunelli@metisprecisionmedicine.org

## Abbreviations

ADCC: Antibody Dependent Cellular Cytotoxicity
APC: AlloPhycoCyanin
CDX: Cell Derived Xenograft
CH: Constant Heavy antibody chain region
CL: Constant Light antibody chain region
ECD: Extra Cellular Domain/Ecto Domain
HGF: Hepatocyte Growth Factor
HRP: HorseRadish Peroxidase
Ig: Immunoglobulin
mAb: Monoclonal Antibody
NSCLC: Non-Small Cell Lung Cancer
OD: Optical Density
PD: Pharmacodynamics
PDX: Patient Derived Xenograft
PK: Pharmacokinetics
TKI: Tyrosine Kinase Inhibitor
TMB: TetraMethylBenzidine
VH: Variable Heavy antibody chain region
VL: Variable Light antibody chain region
