# Correction: hOA-DN30: a highly effective humanized single-arm MET antibody inducing remission of ‘MET-addicted’ cancers

**DOI:** 10.1186/s13046-022-02398-y

**Published:** 2022-05-24

**Authors:** Ilaria Martinelli, Chiara Modica, Cristina Chiriaco, Cristina Basilico, James M. Hughes, Simona Corso, Silvia Giordano, Paolo M. Comoglio, Elisa Vigna

**Affiliations:** 1 Candiolo Cancer Institute, FPO-IRCCS, Strada Provinciale 142, 10060 Candiolo, TO Italy; 2 Department of Oncology, University of Turin, Turin, Italy

**Correction: J Exp Clin Cancer Res 41, 112 (2022)**

**https://doi.org/10.1186/s13046-022-02320-6**

Following publication of the original article [1], author would like to correct the Funding section statement.

Incorrect Funding:

This research was funded by Fondazione AIRC under 5 per Mille 2018 – ID 21052 program - PI: Comoglio PM, GL: Vigna E; by FPRC 5xmille 2014 Ministero Salute to PMC, and by Ministero Salute, Ricerca Corrente 2018–2020. The studies performed by Accelera Srl. were sponsored by Metis Precision Medicine B-Corp.

Correct Funding:

This research was funded by Fondazione AIRC under 5 per Mille 2018 - ID 21052 program - PI: Comoglio PM, GL: Vigna E; **by Italian Association for Cancer Research (AIRC), IG 20210 to S. Giordano, and IG 21770 to S. Corso;** by FPRC 5xmille 2014 Ministero Salute to PMC, and by Ministero Salute, Ricerca Corrente 2018-2020. The studies performed by Accelera Srl. were sponsored by Metis Precision Medicine B-Corp.

The correction does not have any effect on the final conclusions of the paper. The original article has been corrected.
