# Supplementary material for: hOA-DN30: a highly effective humanized single-arm MET antibody inducing remission of ‘MET-addicted’ cancers
Source: J Exp Clin Cancer Res. 2022 Mar 29;41:112. doi: 10.1186/s13046-022-02320-6 (PMC8962049; doi:10.1186/s13046-022-02320-6)
Supplement: Supplementary file 1 — Additional file 1. [file 13046_2022_2320_MOESM1_ESM.pdf]

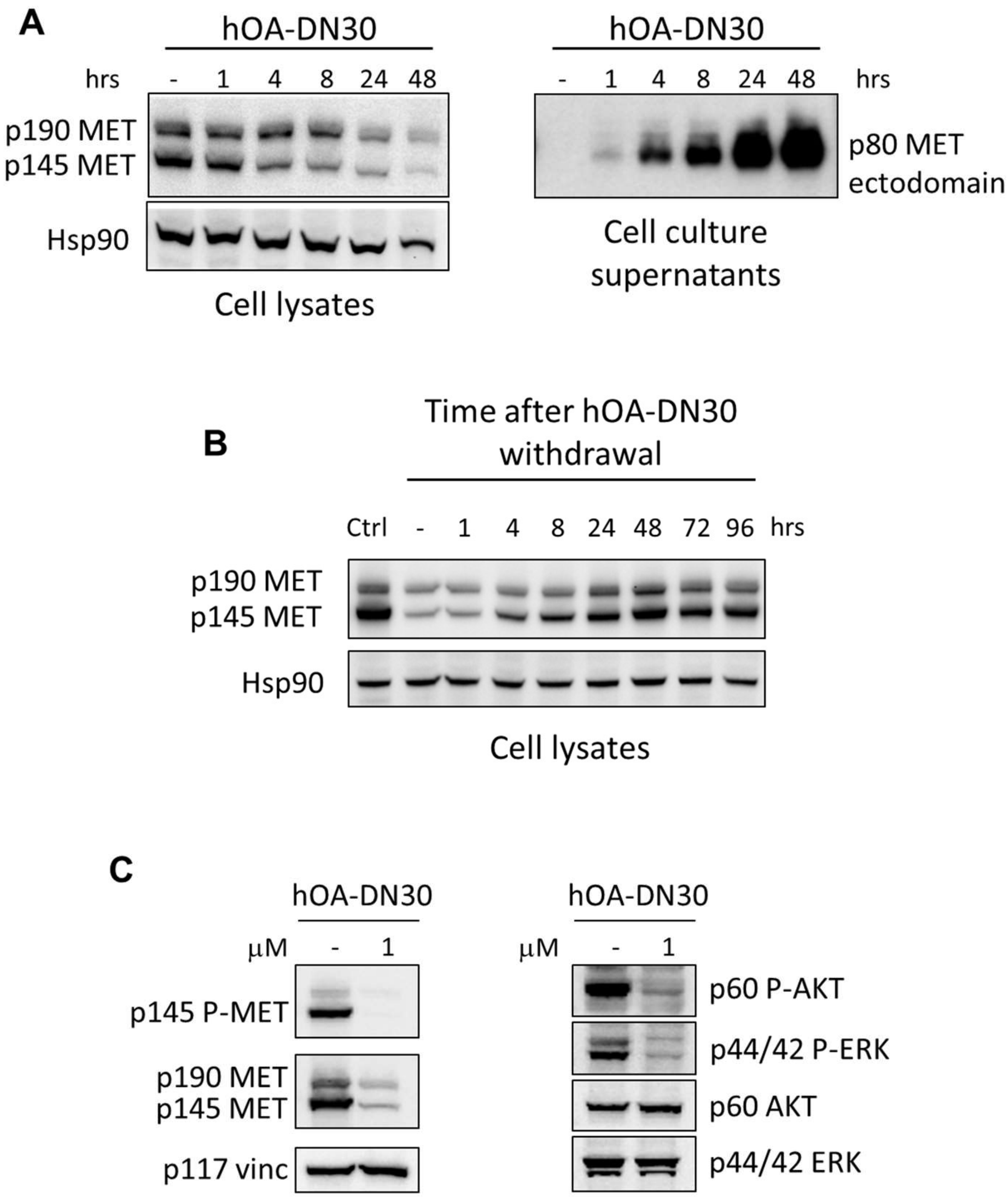

Supplementary Fig. 1. Analysis of hOA-DN30 MET inhibitory properties on EBC-1 MET-addicted NSCLC cells. **a** Kinetic of MET shedding: cells treated with 1μM hOA-DN30 for the indicated times. **b** Recovery of MET expression after shedding: cells treated for 48 hrs and then left untreated for the indicated times. **c** Inhibition of MET and signalling pathway activation. Cells treated for 24 hrs with 1μM hOA-DN30. MET, Phosho-MET, AKT, Phospho-AKT, ERK and Phospho-ERK levels were determined by Western blot analysis of the cell lysates. MET ectodomain levels were determined by Western blot analysis of the cell culture supernatants. To normalize protein loading, filters were re-probed with anti-Hsp90 or anti-vinculin. p145 MET: MET receptor β chain; p145 P-MET: phosphorylated MET receptor β chain; p190 MET: uncleaved MET precursor; p80 MET: soluble MET ectodomain; p60 P-AKT: phosphorylated AKT; p44/42 P-ERK: phosphorylated ERK; p60 AKT: AKT; p44/42 ERK: ERK; p117 vinc: vinculin; Hsp90: Heat shock protein; Data reported in the figure are representative of two experiments.

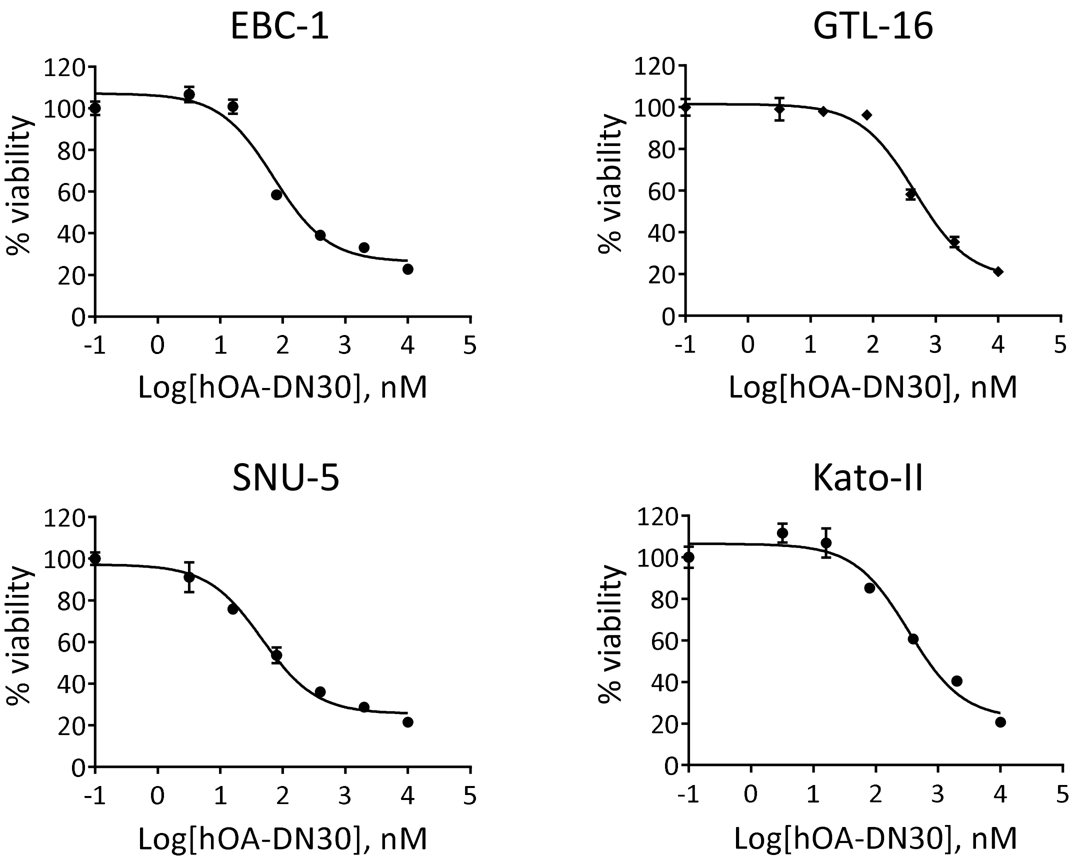

hOA-DN30 growth inhibition analysis

Cell line	Growth inhibition (%)	IC_{50} (nM)	R^2
EBC-1	84.04 ± 7.25	68.01 ± 3.79	0.96
GTL-16	82.90 ± 5.11	372.3 ± 120.1	0.98
SNU-5	79.56 ± 1.51	43.18 ± 4.57	0.98
Kato-II	79.37 ± 0.01	343.90 ± 8.77	0.98

Supplementary Fig. 2. Analysis of viability in MET-addicted cells treated with hOA-DN30. The indicated cancer cell lines have been treated with increasing concentrations of hOA-DN30 for 72 hrs. Determination of viable cells have been done by quantitation of metabolically active cells. Viable cells are reported as percentage of the untreated ones. Each point is the mean of triplicate values; bars represent SD. Graphs are representative of at least two experiments done. The table reports average IC_{50} values calculated from at least two independent experiments and the percentage of maximal inhibition with respect to untreated cells.

A

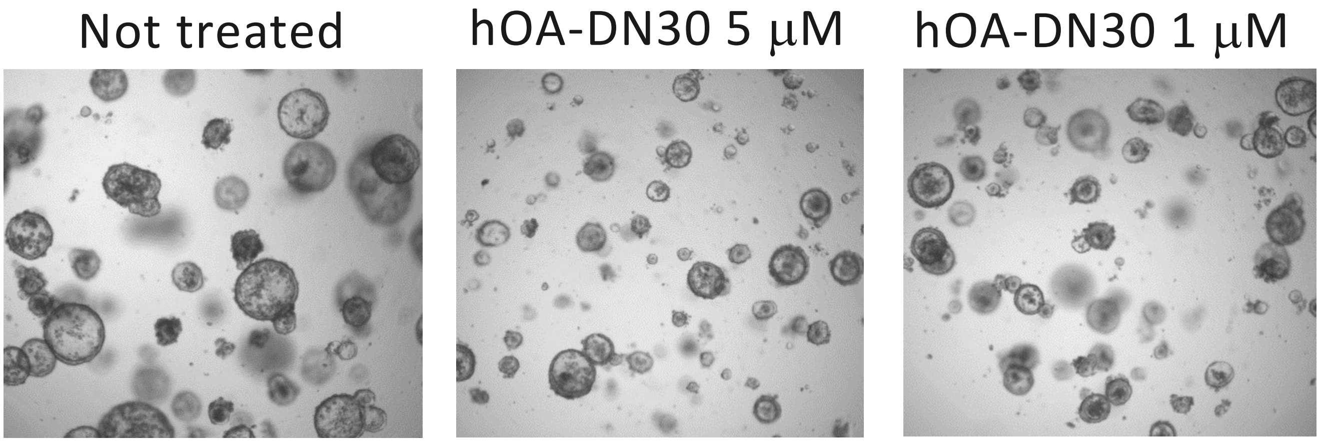

B

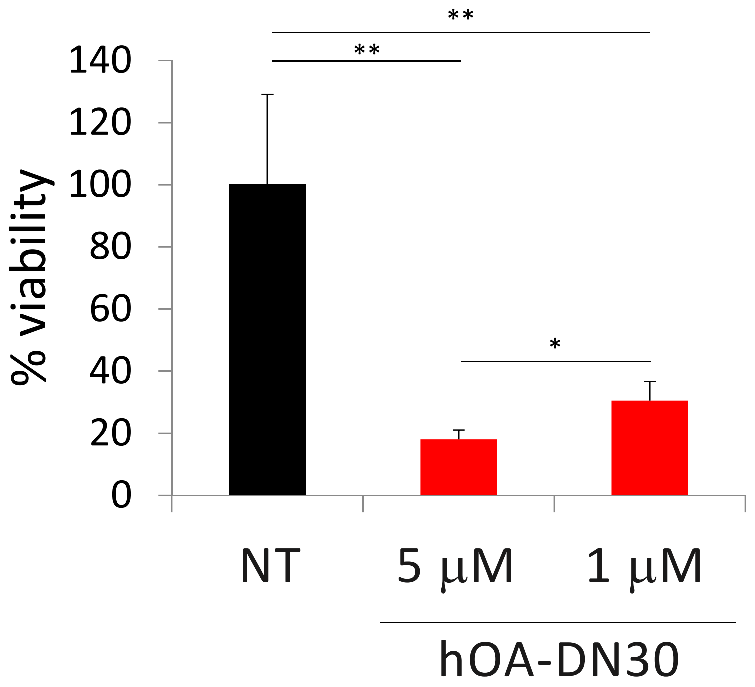

Supplementary Fig. 3. Analysis of viability in tumor organoids derived from a MET-amplified colon cancer tumor (M162) treated with hOA-DN30. **a** Representative images of M162 three dimensional cell culture maintained for 9 days in the presence of the indicated amounts of hOA-DN30. **b** Quantitation of viable cells by Alamar blue staining, reported as percentage of untreated culture (NT). Each point is the mean of triplicate values; bars represent SD. Data reported in the figure are representative of four experiments. Stars indicated *p* values obtained by One-way Anova analysis. **, $p<0.01$; *, $p<0.05$.

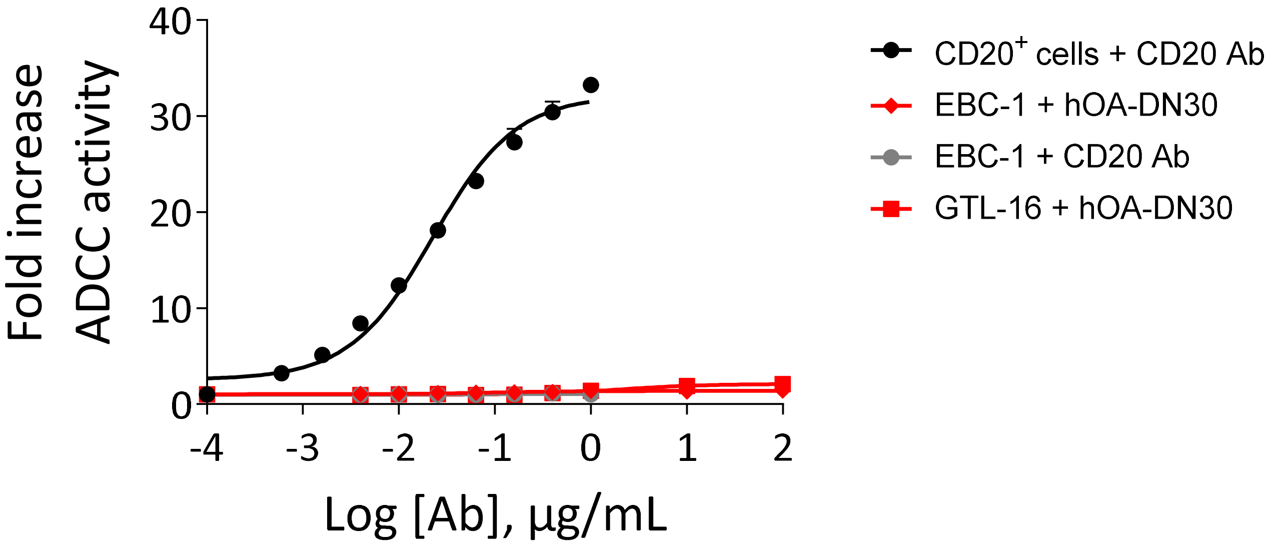

Supplementary Fig. 4. Analysis of ADCC activity induced by hOA-DN30. MET-addicted cells (EBC-1 or GTL-16) were incubated with increasing concentrations of hOA-DN30 for 6 hrs. As positive control CD20 expressing cells were incubated with an antibody able to induce ADCC (CD20 Ab). As control, EBC-1 cells were also incubated with CD20Ab. The graph reports the increase of ADCC activity in the presence of antibodies, with respect to untreated cells. ADCC activity has been determined by ADCC Reporter Bioassay, measuring transcripional activation of NFAT (nuclear Factor of activated T cells) pathway by a luciferase-based reporter system. Each point is the mean of triplicate values; bars represent SD. Data reported in the figure are representative of two experiments.

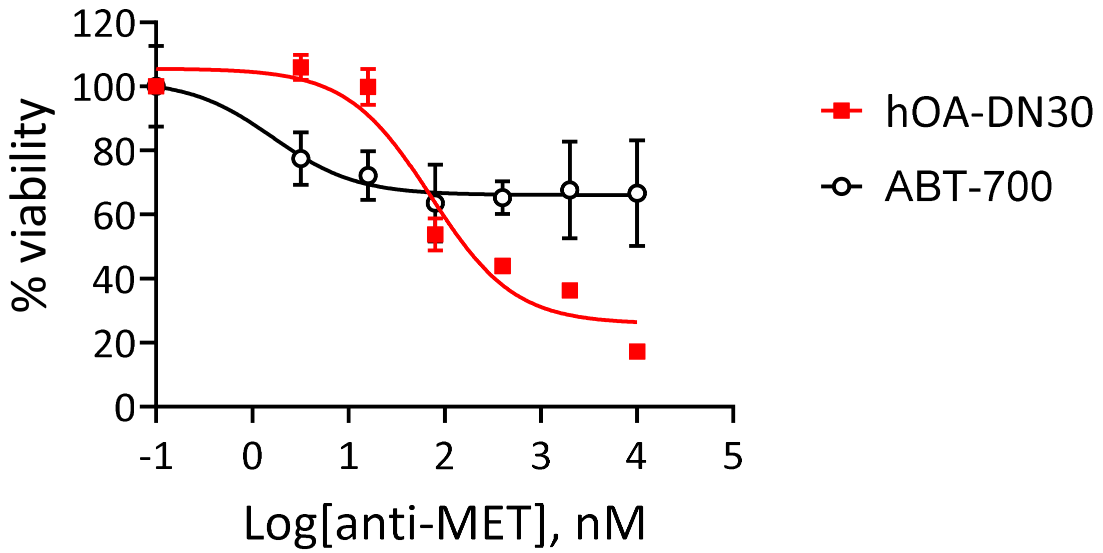

Supplementary Fig. 5. Analysis of viability in MET-addicted EBC-1 cells treated with anti-MET antibodies. Cancer cells have been treated with increasing concentrations of hOA-DN30 or ABT-700 for 72 hrs. Determination of viable cells have been done by quantitation of metabolically active cells. Viable cells are reported as percentage of the untreated ones. Each point is the mean of triplicate values; bars represent SD. Graphs are representative of three experiments done.

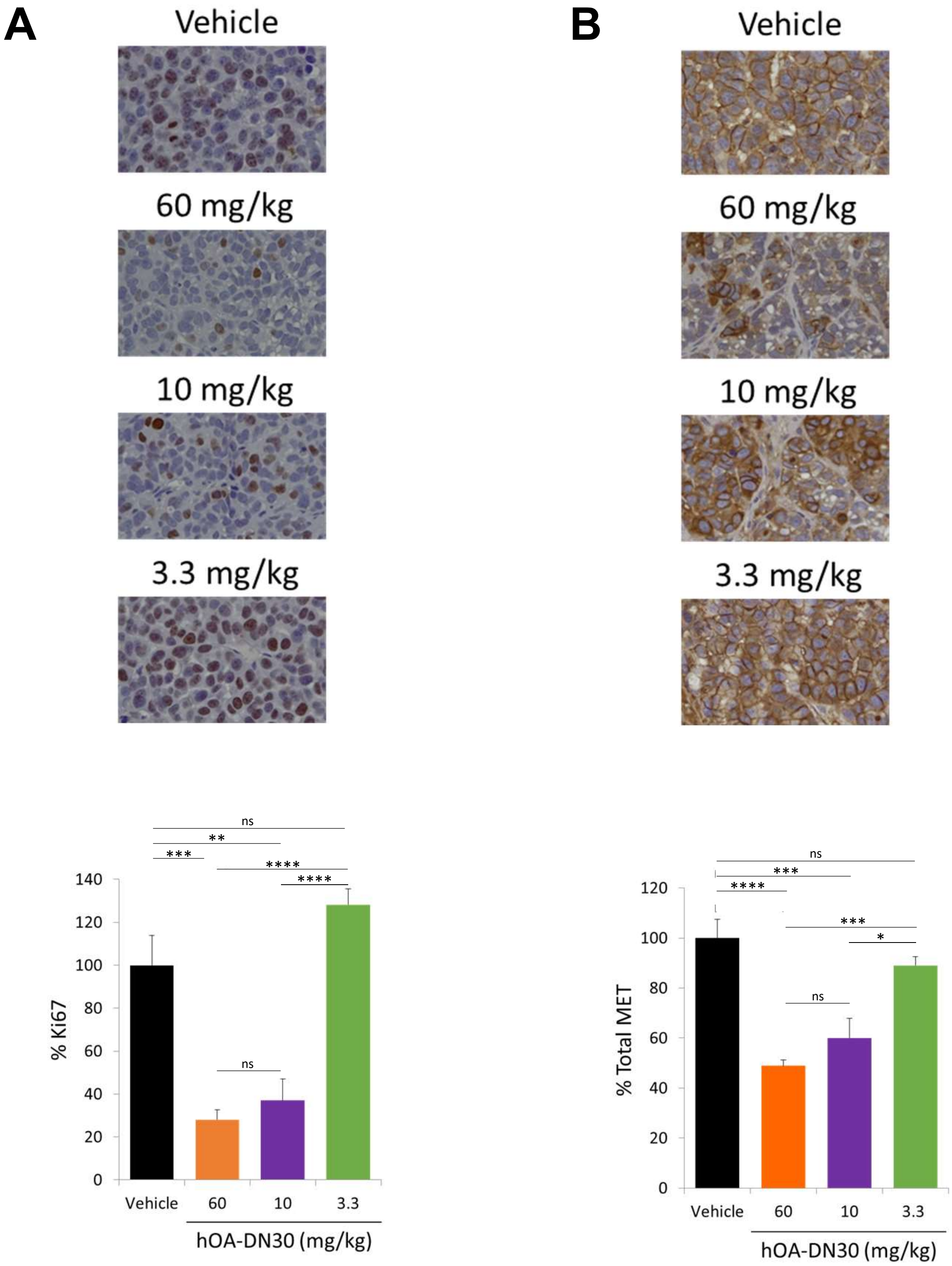

Supplementary Fig. 6. Immunohistochemical analysis of tumors grown in NOD-SCID mice subcutaneously injected with GTL-16 MET-addicted gastric carcinoma cells treated with increasing concentrations (3.3, 10, 60 mg/kg) of hOA-DN30 three times a week. **a** Analysis of tumor cell proliferation by Ki67 staining. Top, representative images; bottom, quantification of the staining represented as percentage with respect to untreated tumors. **b** Analysis of MET expression. Top, representative images; bottom, quantification of the staining represented as fold change with respect to untreated tumors. Bars represent SEM. Stars indicated *p* values obtained by One-way Anova analysis. ****, $p<0.0001$; ***, $p<0.001$; **, $p<0.01$; *, $p<0.05$; ns, $p>0.05$.

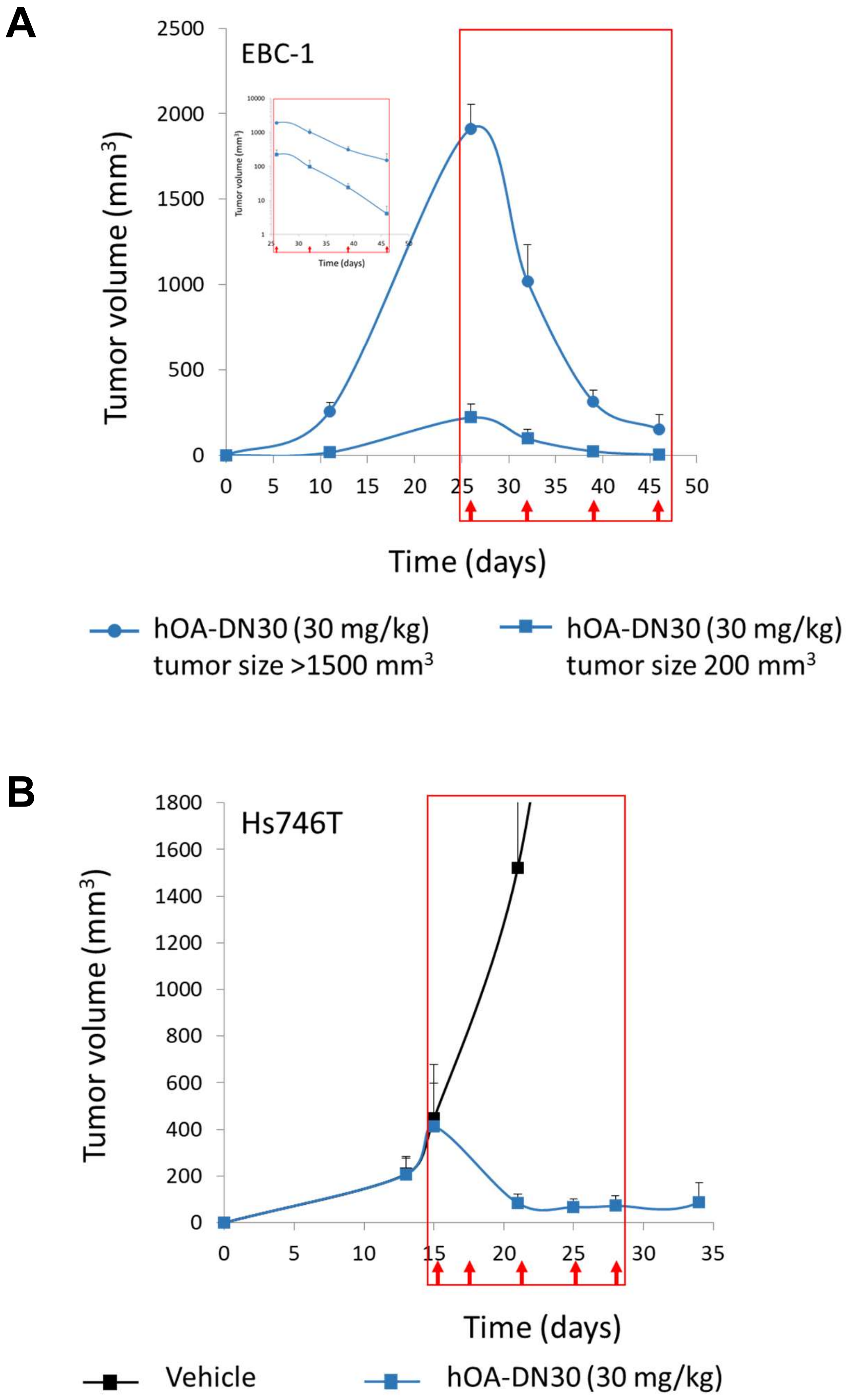

Supplementary Fig. 7. Analysis of hOA-DN30 tumor inhibition *in vivo*. **a** Analysis of tumor growth in NOD-SCID mice subcutaneously injected with EBC-1 MET-addicted gastric carcinoma cells treated with 30 mg/kg of hOA-DN30 once a week. The inset represents tumor volume versus time in Log-Lin scale. **b** Analysis of tumor growth in NOD-SCID mice subcutaneously injected with Hs746T gastric cancer cells treated with 30 mg/kg of hOA-DN30 twice a week. These cancer cells feature amplification and skipping of the exon 14 MET gene. Two-way Anova analysis: Vehicle vs hOA-DN30, $p<0.0001$. Red boxes: period of hOA-DN30 administration; red arrows: antibody deliveries; bars represent SD.

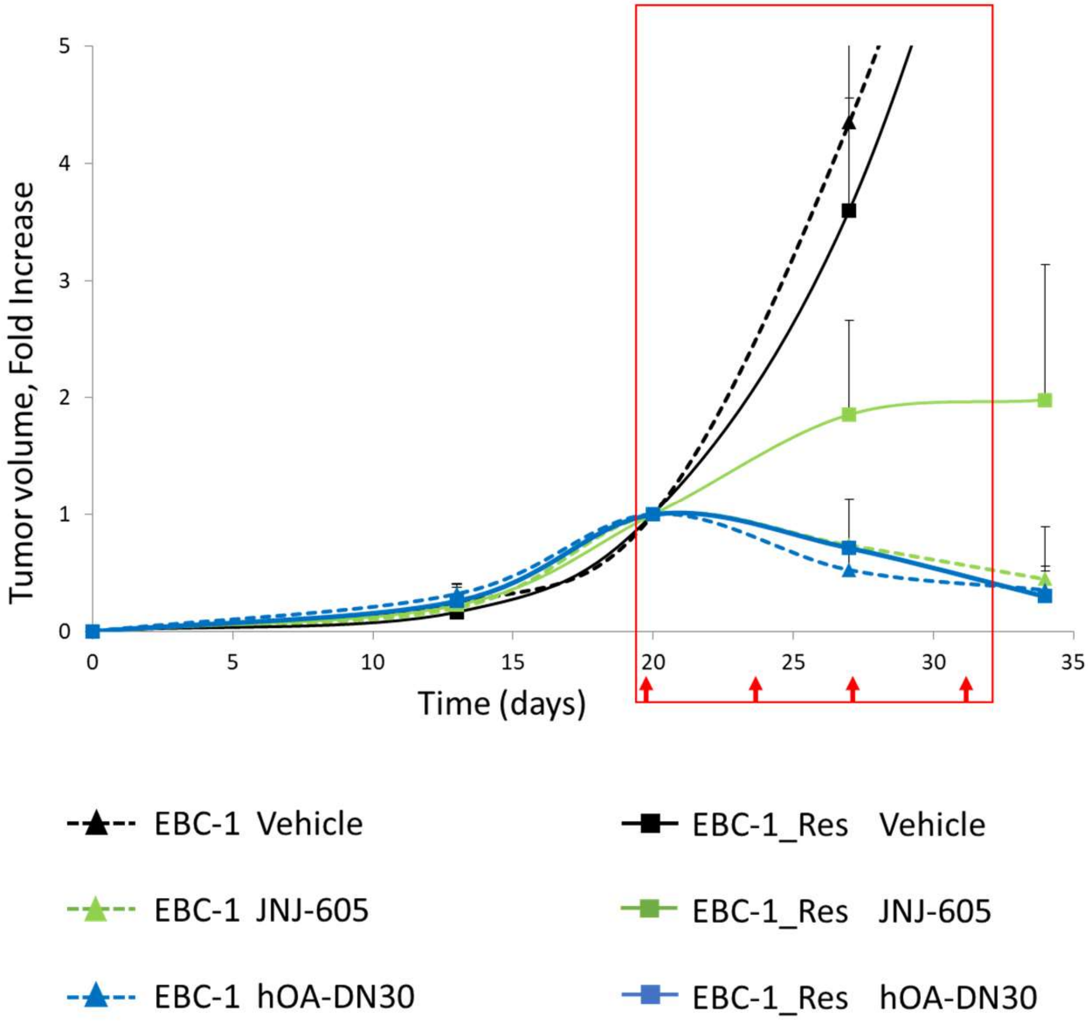

Supplementary Fig. 8. Analysis of hOA-DN30 tumor inhibition *in vivo*. Analysis of tumor growth in NOD-SCID mice subcutaneously injected with EBC-1 MET-addicted lung carcinoma cells or with EBC-1_Res cells featuring a non-cell-autonomous resistance to the MET-specific Tyrosine Kinase Inhibitor JNJ-605. Tumor were treated with 25 mg/kg of JNJ-605 daily, or with 30 mg/kg of hOA-DN30 twice/week. Graph represents fold increase of tumor volume with respect to the volume measured when treatment started (day 20). Red boxes: period of anti-MET molecule administration; red arrows: antibody deliveries; bars represent SD. Two-way Anova analysis: EBC-1: Vehicle vs hOA-DN30, $p<0.0001$; EBC-1_Res: Vehicle vs hOA-DN30, $p<0.0001$; EBC-1: JNJ-605 vs hOA-DN30, $p>0.05$; EBC-1_Res: JNJ-605 vs hOA-DN30, $p=0.004$.

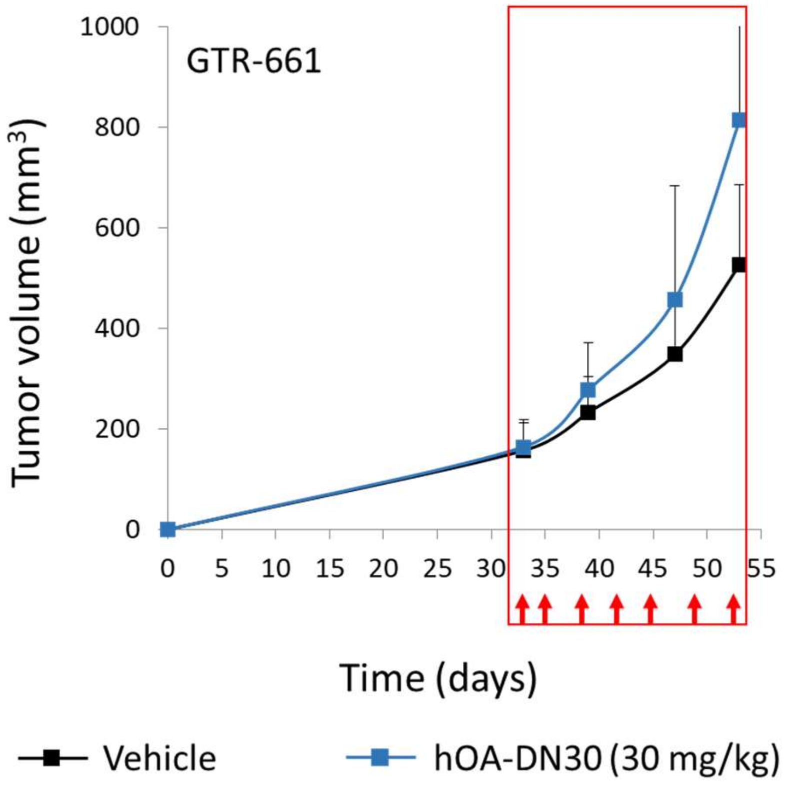

Supplementary Fig. 9. Analysis of tumor growth in NOD-SCID mice subcutaneously implanted with GTR-661 gastric Patient Derived Xenograft treated with 30 mg/kg of hOA-DN30 twice a week. Red boxes indicate the period of hOA-DN30 administration, red arrows antibody deliveries; bars represent SD.

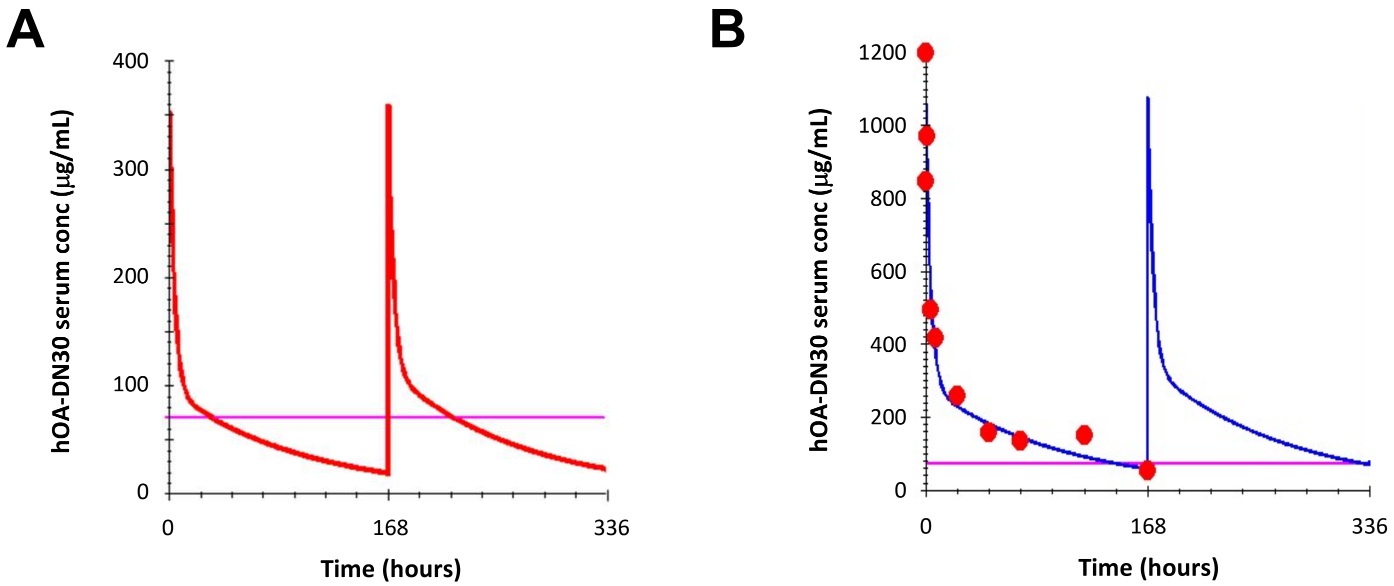

Supplementary Fig. 10. Predicted profile of hOA-DN30 serum levels in NOD-SCID mice bearing EBC-1 tumors. **a** Predicted serum concentrations after intravenous administration of 10 mg/kg hOA-DN30. **b** Predicted serum concentrations after intravenous administration of 30 mg/kg hOA-DN30. Red dots observed concentrations. The pink horizontal line represents the estimated concentration maintaining tumor growth stabilization (Cτ=72.9 mg/mL).

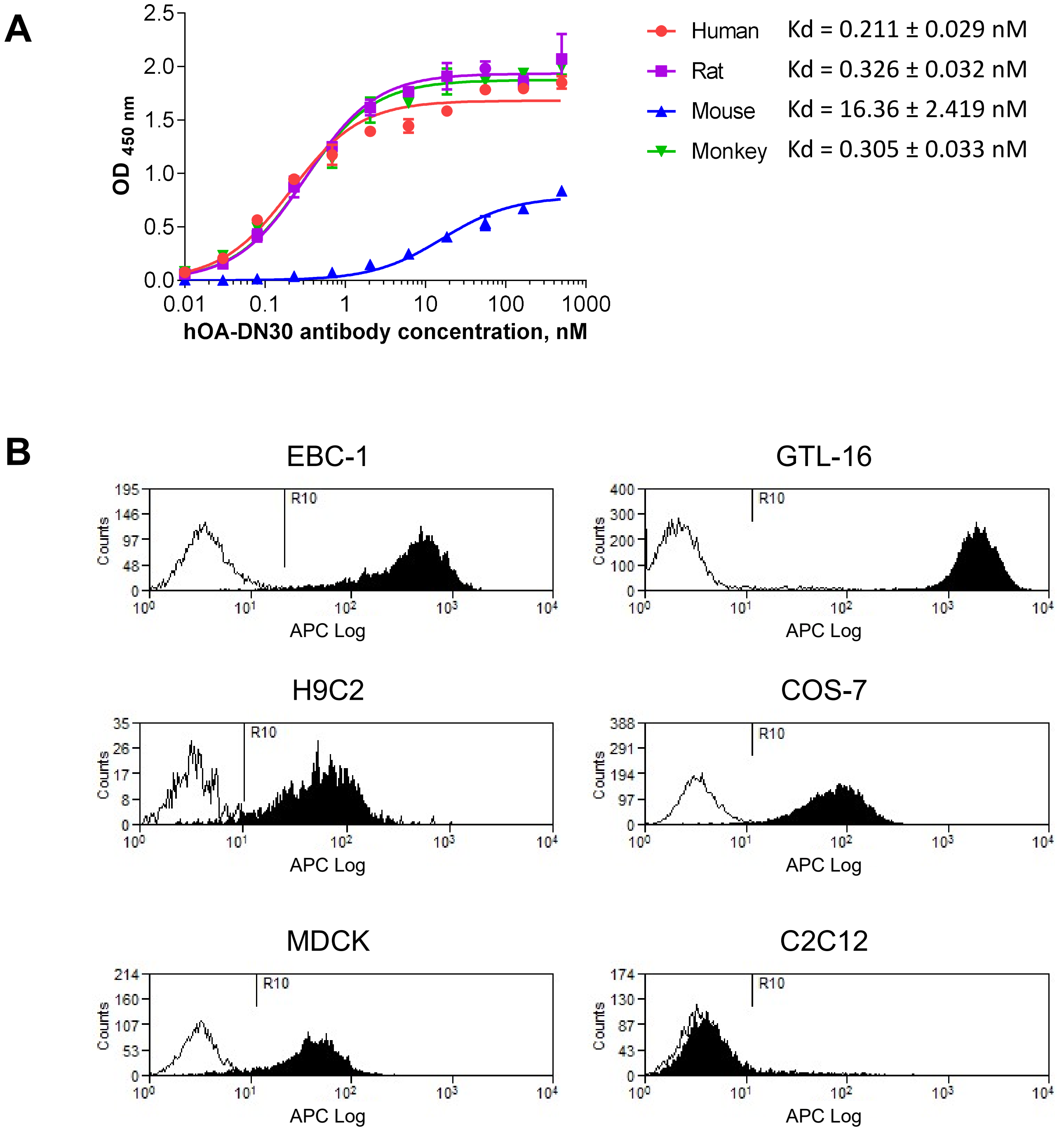

Supplementary Fig. 11. Determination of cross-species reactivity of hOA-DN30. **a** ELISA binding analysis of hOA-DN30 (liquid phase) to purified MET receptor derived from different species (solid phase). O.D., optical density at 450nm. Dissociation constant values (Kd) ± SD are reported in the graph. Each point is the mean of triplicate values; bars represent SD. **b** Cytofluorimeter analysis of hOA-DN30 antibody bound to the surface of MET-expressing cells derived from different species. hOA-DN30 staining in black; anti human IgG (Control) in white. EBC-1: human NSCL Ca cells; GTL-16: human gastric Ca cells; H9C2: rat cardiomyoblasts; Cos-7: African green monkey kidney fibroblast-like cells; MDCK: canine kidney epithelial cells; C2C12: mouse myoblasts. Data reported in the figure are representative of two experiments done.

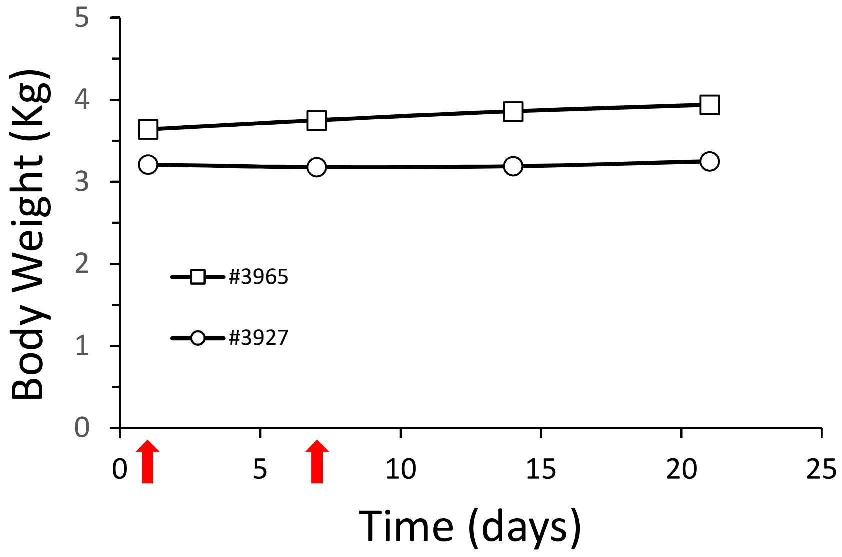

Supplementary Fig. 12. Body weight of the Cynomolgus monkeys during the ‘repeated-dose’ study. Two monkeys (one male - *i.d.* #3965 - and one female – *i.d.* #3927) were included in the experimental group. Animals were administered with 180 mg/kg of hOA-DN30 *i.v.* on day 1 and 7. Red arrows: antibody administrations.
