# Supplementary material for: hOA-DN30: a highly effective humanized single-arm MET antibody inducing remission of ‘MET-addicted’ cancers
Source: J Exp Clin Cancer Res. 2022 Mar 29;41:112. doi: 10.1186/s13046-022-02320-6 (PMC8962049; doi:10.1186/s13046-022-02320-6)
Supplement: Supplementary file 2 — Additional file 2. [file 13046_2022_2320_MOESM2_ESM.docx]

**Supplementary FILE 1**

**MATERIALS AND METHODS**

**hOA-DN30 generation, production and purification**

Humanization of the DN30 mouse antibody has been done by Fair Journey Biologics (Porto, Portugal) using a phage display library approach. Starting from the identification of deviating frame residues (FR), the closest human variable region germline sequences, chosen from germlines with identical canonical fold combination for Complementary Determining Region (CDR) CDR1-CDR2, were selected by an *in silico* analysis. Upon generation of phage display libraries - in which randomization of the variable heavy and variable light regions has been performed - four affinity-driven phage display rounds of selection have been done. At the end of the process, the DN30 humanized variant with the highest affinity for MET between the top 10 antibodies scored by human homology/identity was selected. Variable regions were linked through classical molecular biology techniques to the constant domains sequences derived from human immunoglobulins (Ig), *i.e.* human kappa light chain and IgG1 heavy chain constant domain 1, thus assembling a humanized Fab (Fragment antigen binding ). Conversion of the humanized Fab into a One-Arm single chain antibody (hOA-DN30), was obtained by assembling the antibody light chain, with one full length antibody IgG1 heavy chain and one Fc (Fragment Crystallizable) antibody region in which specific amino acid modifications have been inserted in the CH3 domain, to produce the ‘knob into hole’ structure (1). The heavy chain (VH-CH1-CH2-CH3) includes the 389T🡪W (Knob mutation), the Fc (CH2-CH3 polypeptide), includes the replacement of three amino acids: 389T🡪S; 391L🡪A; 438Y🡪V (Hole mutations). The three cDNAs corresponding to the above described antibody chains (VL-CL; VH1-CH1-CH2-CH3; CH2-CH3) were expressed into eukaryotic cells (ExpiCHO-S cells) and one-arm antibodies were purified from cell culture supernatants by a two steps chromatography procedure including a passage on Hitrap MabSelect Sure column followed by a second step on Superdex 200 26/600 size exclusion column. Purity and correct molecular size of the product were assessed by SDS-PAGE analysis followed by Gel Code Blue staining (Thermo Fisher Scientific).

**Cell culture**

A549, NCI-H226, NCI-H441 (all human lung carcinoma cells), HPAF-II human pancreatic adenocarcinoma cells, SNU-5, Hs746T (both human gastric carcinoma cells), H9c2 rat myoblast cells, C2C12 mouse myoblast cells, MDCK dog kidney cells, COS-7 monkey kidney cells were from ATCC/LGC Standards S.r.l (Sesto San Giovanni, Italy). EBC-1 human lung carcinoma cells were from the Japanese Collection of Research Bioresources (Osaka, Japan). Kato-II human gastric carcinoma cells were a kind gift of Y. Zimmer, University of Bern. GTL-16 were derived from MKN-45 human gastric carcinoma cells (2). Cells were maintained as recommended by the supplier. M162 colon cancer organoids were derived from tumor material of a patient resistant to EGFR target therapy (3) propagated in mice and maintained as described in (4).

**ELISA assays**

For affinity determination, pure recombinant MET extracellular domain from different species (human, mouse, rat, or monkey) fused in frame with a human Fc domain (R&D System, Minneapolis, MN) (100 ng/well) was in solid phase and pure MvDN30 or hOA-DN30 (range of dilution: 0-500 nM) were in liquid phase.Revealing was done with HRP-conjugated anti-human k light chain antibody (Sigma Life Science, St Louis, MO). After addition of TMB substrate (Sigma Aldrich), the signal from the colorimetric reaction was quantified by the multi-label reader VICTOR X4 (Perkin Elmer Instrument INC., Whaltman, MA).

For hOA-DN30 quantification in mouse serum a goat anti-human IgG (γ-chain specific) antibody (1 μg/mL, Sigma Aldrich) was directly adsorbed in solid phase. Pure hOA-DN30 (calibration curve, range 25-25000 ng/mL) or serum samples were in liquid phase. For determination of hOA-DN30 in monkey serum samples, neutravidin-coated plates are used to immobilize biotin-labeled goat anti-human IgG monkey adsorbed antibody (0.2 μg/mL, Southern Biotech). Pure hOA-DN30 (calibration curve, range 25 - 4800 ng/mL) or serum samples were in liquid phase. Revealing and colorimetric assay were done as described above. The concentration of hOA-DN30 in serum was achieved by interpolating the optical density (OD) readings of the samples with the calibration curve fitted by a weighted (1/Y) 4 parameters regression function.

**Antibody binding on the cell surface**

The indicated cells, detached with PBS-EDTA 1M, were stained with hOA-DN30 (1 mg/ml) 30’ on ice. Cell surface bound hOA-DN30 antibodies were revealed with APC-conjugated mouse anti-human IgG (BD Biosciences). Fluorescent signal was analyzed by Summit 4.3 software (Dako, Santa Clara, CA, USA). Signal derived from cells incubated only with the secondary antibody was set as: 0 < MFI < 10^1^. Signal was considered positive when MFI > 10^1^.

**MET shedding and MET inhibition**

For dose-dependent experiments, sub-confluent A549 cells were incubated in serum-free medium for 48 hrs with the indicated concentration of hOA-DN30 (range 0.037-1 µM). For time-dependent experiments, sub-confluent A549 or EBC-1 cells were incubated in serum free medium in the presence of hOA-DN30 (1 µM). Samples (cell culture supernatants and cell lysates in Laemmli buffer) were collected at different time points, from 1 hr to 6 days. (15 µg of cell extracts and 15 µl of cell culture supernatants were resolved by SDS-PAGE and analyzed by Western blotting. For the analysis of MET recovery after shedding, sub-confluent A549 or EBC-1 cells were incubated in serum-free medium in the presence of hOA-DN30 (1 µM); after 48 hrs, cell monolayers were extensively washed with PBS and fresh serum-free medium was added. Cell culture supernatants and cell lysates were collected at different time points (from 1 hr to 4 days), and analyzed as described above.

For inhibition of MET and downstream signaling pathway activation, GTL-16 or EBC-1 cells were treated with the indicated concentrations of hOA-DN30 for 24 hrs in serum free media. Cell monolayers were lysed and analysed as described above.

**Western Blotting**

Primary antibodies for Western blot detection were: anti-human Met mAb (3D4 Invitrogen Corporation, Camarillo, California), anti-human Met DL21 mAb recognizing Met β chain extracellular domain (Vigna et al., 2008), anti p-Met Tyr1234/1235 (D26), anti-pAKT Ser473, anti-AKT, anti-pERK1/2 Thr1234/1235, anti-ERK1/2, anti HSP90 polyclonal Abs (all from Cell Signaling Technology, Beverly, MA); anti-Vinculin clone hVIN-1 (Sigma Life Sciences). HRP-conjugated secondary antibodies were from GE Healthcare (Freiburg, Germany).

**Biological assays**

For scatter assay, HPAF-II cells were seeded in a 96 well plate (8000 cells/well). After 24 h cells were treated with HGF (8 ng/ml), hOA-DN30 (200 nM), or DN30 mAb (200 nM); after 24 hrs cells were stained with crystal violet (Sigma-Aldrich, St Louis, MO). Cell scattering was determined by optical microscopy.

For cell growth assay, cells were seeded in a 96 well plate (2000 cells/well). After 24 h cells were treated with increasing concentration (from 0.0032 to 10 µM) of hOA-DN30. Cell viability was evaluated after 72 h by CellTiter-Glo luminescent cell viability assay (Promega Corp., Madison, WI), according to the manufacturer’s instructions. Chemiluminescence was detected with VICTOR X4.

For proliferation assay, sub-confluent cells were treated with hOA-DN30 (1 µM). After 48 hrs cellular DNA synthesis was determined measuring EdU incorporation using the Click-iT® EdU Alexa Fluor® 647 Flow Cytometry Assay Kit (Thermo Fisher Scientific) according to manufacturer’s instruction.

For cell death assay, cells were seeded and treated as described above in cell growth assay. Cytotoxicity was evaluated after 72 hrs by CellTox™ Green Cytotoxicity Assay (Promega Corp., Madison, WI), according to the manufacturer’s instructions. Fluorescence was detected with VICTOR X4 and data normalized over CellTiter-Glo luminescence.

For determination of tumor organoids viability, M192 cells plated with matrigel in 96 well plate were incubated for 5 hrs with Alamar blue (Life Technologies) diluted 1:10. The signal measured in the cell culture supernatants by VICTOR X4 was set as time 0, then freshly prepared medium with 1 or 5 μM hOA-DN30 was added on the culture. Every three days, medium was replaced with fresh one containing the antibody. After nine days, a new assessment of Alamar blue signal was performed. Cell growth was measured calculating the ratio between day 9 and day 0 for each sample.

For determination of Antibody Dependent Cellular Cytotoxicity (ADCC), EBC-1 cells were seeded in a 96 well plate (10000 cells/well). After 24 h cells were treated with increasing concentration (from 0.004 to 100 µg/mL) of hOA-DN30 and effector cells were added. After 6hrs, ADCC activity has been measured by ADCC Reporter Bioassay (Promega Corp) according to the manufacturer’s instructions.

**Evaluation of tumor growth inhibition *in vivo***

All procedures in mice were performed according to protocols approved by the Ethical Committee for animal experimentation of the Candiolo Cancer Institute and by the Italian Ministry of Health.

To generate Cell Derived Xenografts (CDX), cancer cells were resuspended in 200 µl Iscove Modified Dulbecco Medium (IMDM, Sigma Life Science) (GTL-16 and EBC-1) or in 100 μl Iscove + 100 μl Matrigel matrix (Corning Inc.) (SNU-5 and Hs746T). Cells were then injected subcutaneously into the right posterior flank of adult NOD-SCID mice. The number of injected cells/mouse was: GTL-16, 0.9 x 10^6^; EBC-1, 1.5 x 10^6^; SNU-5, 5 x 10^6^; Hs746T, 3 x 10^6^. When tumors were established, sizes were checked and the animals were divided in experimental arms homogeneous for tumor size and randomly assigned to the different treatments. hOA-DN30 was administered by intravenous injection. Tumor size was evaluated periodically with a caliper. Tumor volume was calculated as described (5).

For evaluation of hOA-DN30 dose-response in GTL-16, 7 days after cell injection mice were divided into 5 groups (*n*=6; average tumor volume: 79.3 ± 34.6 mm^3^). Starting from day 8 mice were treated 3xweek by tail vein injection with different doses of hOA-DN30 (60-10-3.3 mg/kg); one group received PBS (vehicle). At the end of the experiment (24^th^ day after cell injection), tumors were extracted, fixed and paraffin-included for immunohistochemical analysis. Antibodies used for the staining were: anti-human Ki67 clone MIB-1 mAb (Dako); AF276 anti-human MET and AF2480 anti-human/mouse phosphor-MET(Tyr 1234-1235) polyclonal antibodies (R&D System). For each tumor, at least 5 different slides were analyzed.

For evaluation of hOA-DN30 dose-response in EBC-1, 12 days after cell injection (average tumor volume 76.1 ± 46.9 mm^3^), mice were divided into 5 groups and treated once a week by tail vein injection as follows: hOA-DN30 5 mg/kg (*n*=4), hOA-DN30 10 or 30 mg/kg (*n*=6), and Vehicle (PBS, *n*=6). Animals were treated until day 32; mice that received 10 or 30 mg/kg hOA-DN30 were left untreated and tumor growth was monitored for further 14 or 21 days, respectively.

For evaluation of hOA-DN30 efficacy on tumors of different size, twenty-six days after EBC-1 cell injection, two experimental groups were generated: one group included mice with very large tumors (average tumor volume: 1911.7 ± 144.3 mm^3^; *n*=2) and the other group smaller tumors (average tumor volume: 222.3 ± 79.8 mm^3^; *n*=5). Mice were treated with hOA-DN30 (30 mg/kg) once a week, until day 46.

For evaluation of hOA-DN30 schedule, EBC-1 tumors were generated as described above. After 11 days mice were randomized in three groups (average tumor volume: 222.4 ± 147.6 mm^3^). Three days after randomization, 2 groups (*n*=6) were treated with hOA-DN30 (60 or 10 mg/kg); one group (*n*=8) received PBS (vehicle). hOA-DN30 administrations were done with different schedule, delivering to the animals the same total amount of antibody (2400 µg/mouse). At each injection mice received: group A, hOA-DN30 60 mg/kg (1200 µg/injection) on day: 14 and 28; group B, hOA-DN30 10 mg/kg (200 µg/injection) on day: 14, 16, 18, 21, 23, 25, 28, 30, 32, 35, 37, 39; group C, Vehicle (PBS) as group B. Tumor growth was monitored until day 43.

For evaluation of hOA-DN30 efficacy on SNU-5, 36 days post- cell injection mice were divided into 2 groups (*n*=9; average tumor volume: 217.6 ± 120.3 mm^3^). One group received hOA-DN30 (30 mg/kg) 2xweek, the other received PBS (vehicle). Mice were treated until day 74, and then were left untreated. Tumor growth was monitored until day 99.

For evaluation of hOA-DN30 efficacy on Hs746T, 15 days post- cell injection, mice were divided into 2 groups (*n*=6; average tumor volume: 403.7 ± 170.3 mm^3^). One group received PBS (vehicle), while the other group received hOA-DN30 30 mg/kg 2xweek. Animals were treated until day 28 and then were left untreated. Tumor growth was monitored until day 34.

For evaluation of hOA-DN30-induced MET shedding *in vivo*, mice carrying SNU-5 tumors (average tumor volume 1378.9 ± 519.6) were divided in 2 groups: one was treated with hOA-DN30 (30 mg/kg, *n*=5), while the other received PBS (Vehicle, *n*=2). As control, one tumor-free mouse was injected with hOA-DN30. Animals were bled at day 0 and 24, 48, 72, 144 hrs after treatment. Met ECD concentrations in serum samples were determined by ELISA. AF276 anti-MET antibody (0.5 μg/mL) was in solid phase. Pure MET-ECD (Basilico et al., Int J can 2018) (calibration curve, range: 0.00013-100 nM) or serum samples were in liquid phase. MET-ECDs bound to the solid phase were revealed with DO24 anti-MET antibody (1 μg/mL) (6), plus HRP- conjugated secondary antibodies goat anti-mouse IgG (1:5000, Jackson Immunological). Colorimetric assay was done as described above. The concentration of MET-ECD in serum was achieved by interpolating the OD readings of the samples with the calibration curve in the linear range.

To generate Patient Derived Xenografts (PDX), tumor materials derived from human gastric tumor specimens expanded for at least 2 generations in mice were implanted in a subcutaneous pocket generated in the flank of NOD-SCID mice as described in (7). When tumors were established, sizes were checked and the animals were divided in groups, homogeneous for tumor size. Mice were assigned randomly to the different experimental arms. hOA-DN30 was administered systemically by intravenous injection. Tumor volume was determined as described for CDXs.

For evaluation of hOA-DN30 efficacy on GTR-661, 33 days post- implant mice were divided into 2 groups (*n*=9; Average tumor volume: 161.1 ± 52.5 mm^3^). One group received hOA-DN30 (30 mg/kg) 2xweek, the other received PBS (vehicle), until day 53.

For evaluation of hOA-DN30 efficacy on GTR-561, 30 days post- implant mice were divided into 2 groups (Average tumor volume: 109.3 ± 50.2 mm^3^). One group received vehicle (*n*=7), while the other received hOA-DN30 (*n*=6), until day 63, and then were left untreated. Tumor growth was monitored until day 160.

For evaluation of hOA-DN30 efficacy on SG-16, 49 days post- implant mice were divided into 2 groups (*n*=4; Average tumor volume: 183.3 ± 41.9 mm^3^). One group received vehicle, and the other group received hOA-DN30 (30 mg/kg) 2xweek, until day 71, and then were left untreated. Tumor growth was monitored until day 120.

**MET gene copy number determination**

Genomic DNA from cell lines or PDX specimen was obtained by Maxwell RSC® Cell DNA purification kit and Tissue DNA kit (Promega), respectively. MET gene copy number was determined by real-Time qPCR using the taqman probe Hs04993403_cn (Thermo Fisher Scientic). To normalize gDNA in the samples RNAase-P taqman probe Hs00468130_cn (Thermo Fisher Scientific) was used.

**Pharmacokinetics and toxicological analysis**

For pharmacokinetic (PK) evaluation in mice, adult male NOD/SCID mice bearing or not tumors were used. Tumors were generated by subcutaneous injection of EBC-1 cells as escribed above. After 4 weeks animals were randomly assigned to 6 experimental groups homogeneous for tumor size (average tumor volume: 188.3 ± 53.6 mm^3^): group A: *n*=2; groups B-F: *n*=4. Animals received 30 mg/kg of hOA-DN30 in a single intravenous administration. Blood was taken at pre-dose (time 0, group A), 0.083 hr (group B), 0.5 hr (group C), 1 hr (group D), 4 hrs (group E), 8 hrs (group F), 24 hrs (group C), 48 hrs (group D), 72 hrs (group F), 120 hrs (group E) and 168 hrs (group D) post-dosing. hOA-DN30 levels in mouse serum were determined by ELISA assay (see above). PK analysis was performed according to standard non-compartmental and compartmental approach using Phoenix-WinNonlin package (v. 6.3, Pharsight Inc, Certara Company, USA) by Accelera S.r.l. (Nerviano, Italy).

For PK evaluation in monkeys, the experiments have been performed by Accelera S.r.l.. All the animal procedures (including housing, health monitoring, restrain, dosing, etc.) and ethical revision were performed according to the current Italian legislation (Legislative Decree March 4th, 2014 n. 26) enforcing the 2010/63/EU Directive on the protection of animals used for biomedical research. Three adult male cynomolgus monkeys were administered with hOA-DN30 as a single intravenous bolus at the dose of 11 mg/kg. Serum levels of hOA-DN30 were evaluated after pre-dose, 5 min, 30 min, 1, 4, 8, 24, 48, 72, 168, 240, 336, 504 and 672 hours post-dosing. Compound concentrations in serum were determined by ELISA assay (see above). PK analysis was performed according to standard non-compartmental approach as described above.

Determination of hOA-DN30 tolerability was performed by Accelera S.r.l.. The antibody was administered as intravenous infusion, to two adult Cynomolgus Monkeys (one male, one female). During the dose-escalating study, hOA-DN30 was delivered according to ascending doses (30, 90 and 180 mg/kg), one week apart, while during repeated dose analysis, the dose of 180 mg/kg was administered at weekly interval for two times. Animal’s healthy status was monitored at least once daily during the pre-test period; at least twice daily on treatment. Clinical signs observation included, but are not limited to, behaviour and activity, appearance, major physiological functions. Body weights were measured pre-test, on dosing day, and then once a week. Food consumption was evaluated daily. Blood was collected pre-test, and before each dose change to determine standard haematological and serum chemical parameters. At necropsy, body weight measurement and macroscopic examinations were done.

**Pharmacodynamics analysis**

Adult NOD/SCID mice subcutaneously injected in the flank with 1.5x10^6^ EBC-1 cells were administered with hOA-DN30 intravenously once a week for four weeks at the doses of 5, 10, and 30 mg/kg. The analysis was performed by Accelera S.r.l., applying an E_max_ (maximum kill rate) PK/PD model (8) to tumor volumes. The threshold concentration for tumour stabilization (C_τ_) was calculated from the equilibrium status of the first differential equation of the model.

**Statistical analysis**

Averages, standard deviations and *P* values obtained by Student’s t Test were calculated using Microsoft Office Excel 2010 software (Microsoft Corporation, Redmond, Washington). To calculate Kd and Bmax, data from ELISA assay were analyzed and fitted according to nonlinear regression, one site binding hyperbola curve, using GraphPad Prism software (GraphPad Software, San Diego, California). To calculate IC_50_, data from growth assays were analyzed and fitted according to a nonlinear regression, sigmoidal dose response curve, using GraphPad Prism software. *P* values obtained by One-way or Two-way Anova were calculated using GraphPad Prism software.

**References**

1. Ridgway JB, Presta LG, Carter P. 'Knobs-into-holes' engineering of antibody CH3 domains for heavy chain heterodimerization. Protein Eng. 1996;9(7):617-21.

2. Rege-Cambrin G, Scaravaglio P, Carozzi F, Giordano S, Ponzetto C, Comoglio PM, et al. Karyotypic analysis of gastric carcinoma cell lines carrying an amplified c-met oncogene. Cancer Genet Cytogenet. 1992;64(2):170-3.

3. Bardelli A, Corso S, Bertotti A, Hobor S, Valtorta E, Siravegna G, et al. Amplification of the MET receptor drives resistance to anti-EGFR therapies in colorectal cancer. Cancer Discov. 2013;3(6):658-73.

4. Martin V, Chiriaco C, Modica C, Acquadro A, Cortese M, Galimi F, et al. Met inhibition revokes IFNγ-induction of PD-1 ligands in MET-amplified tumours. Br J Cancer. 2019;120(5):527-36.

5. Pacchiana G, Chiriaco C, Stella MC, Petronzelli F, De Santis R, Galluzzo M, et al. Monovalency unleashes the full therapeutic potential of the DN-30 anti-Met antibody. J Biol Chem. 2010;285(46):36149-57.

6. Prat M, Crepaldi T, Pennacchietti S, Bussolino F, Comoglio PM. Agonistic monoclonal antibodies against the Met receptor dissect the biological responses to HGF. J Cell Sci. 1998;111 ( Pt 2):237-47.

7. Migliardi G, Sassi F, Torti D, Galimi F, Zanella ER, Buscarino M, et al. Inhibition of MEK and PI3K/mTOR suppresses tumor growth but does not cause tumor regression in patient-derived xenografts of RAS-mutant colorectal carcinomas. Clin Cancer Res. 2012;18(9):2515-25.

8. Simeoni M, Magni P, Cammia C, De Nicolao G, Croci V, Pesenti E, et al. Predictive pharmacokinetic-pharmacodynamic modeling of tumor growth kinetics in xenograft models after administration of anticancer agents. Cancer Res. 2004;64(3):1094-101.
