# Supplementary material for: hOA-DN30: a highly effective humanized single-arm MET antibody inducing remission of ‘MET-addicted’ cancers
Source: J Exp Clin Cancer Res. 2022 Mar 29;41:112. doi: 10.1186/s13046-022-02320-6 (PMC8962049; doi:10.1186/s13046-022-02320-6)
Supplement: Supplementary file 3 — Additional file 3. [file 13046_2022_2320_MOESM3_ESM.pdf]

Supplementary Table 1: MET gene copy number

Cell line or PDX	Fold Amplification[#]
A549	1 ± 0.06
EBC-1	11.9 *
GTL-16	10.42 ± 0.69
SNU-5	9.2 *
Kato-II	18[§]
Hs746T	17.9*
Caki-I	2.14 ± 0.04
NCI-H226	1.13 ± 0.07
NCI-H441	2.37 ± 0.15
GTR-661	3.11 ± 0.61
GTR-561	74.66 ± 5.89
SG16	12.63 ± 0.28

[#] Fold change in MET gene copy number with respect to the diploid cell line A549, determined by real-time qPCR.
* Data from Pennacchietti et al., Can Res 2014; PMID: 25217525
[§] Data from Smolen et al., PNAS 2006; PMID: 16461907

Supplementary Table 2: hOA-DN30 pharmacokinetics parameters in the mouse. Compartmental analysis results

Parameter	mice w/o tumor	Mice with tumor
T1/2 α (h)	3.37	2.71
T1/2 β (h)	120.60	72.05
Cmax (μg/ml)	1142.09	1055,18
AUC (h*μg/ml)	70728.72	32934.53
Vss (ml/kg)	70.07	86.40
CL (ml/h/kg)	0.424	0.911
CLD (ml/h/kg)	3.205	4.460
V1 (ml/kg)	26.27	28.43
V2 (ml/kg)	43.80	57.97

T1/2,α: Half-life of the first phase; T1/2,β: Half-life of the second phase; Cmax: maximum concentration; AUC: Area Under serum concentration; CL: Serum Clearance; CLD: Distribution Clearance; V1: Volume of the central compartment; V2: Volume of the peripheral compartment.

Supplementary Table 3: hOA-DN30 pharmacokinetics parameters in the mouse. Non-compartmental analysis results

Parameter	**Mice w/o tumor**	**Mice with tumor**
C_{max} (μg/ml)	1188.55	1200.27
T_{max} (h)	0.083	0.083
C_{last} (μg/ml)	145.79	52.47
T_{last} (h)	168	168
AUC_{last} (h*μg/ml)	46313.90	30557.25
AUC∞ (h*μg/ml)	70055.70	35109.08
AUC Extrapol (%)	33.89	12.96
CL (ml/h/kg)	0.428	0.854
Vss (ml/kg)	65.93	71.27
Vz (ml/kg)	69.74	74.12
MRT_{last} (h)	63.29	57.89
MRT∞ (h)	153.96	83.41
T 1/2.z (h)	112.88	60.13

C_{max}: maximum concentration; T_{max}: time to peak concentration; C_{last}: last detectable concentration; T_{last}: Time to last detectable concentration; AUC_{last}: Area under serum concentration vs. time curve up to the last detectable concentration; AUC∞: Area under serum concentration vs. time curve up to infinite time; AUC Extrapol: percentage of the area under the curve extrapolated to infinity; CL: Serum Clearance; Vss: Volume of distribution at steady-state; Vz: Volume of distribution on the terminal phase; MRT_{last}: Mean Residence Time at the time of the last detectable concentration; MRT∞: Mean Residence Time up to infinite time; T1/2,z: Terminal half-life.

Supplementary Table 4: hOA-DN30 pharmacokinetics parameters in the monkey. Non-compartmental analysis results

Parameter	Mean (*n*=3)	S.D.	%CV
C_0 (µg/mL)	393	20.8	5.3
AUC_{last} (µg*h/mL)	31200	1960	6.3
AUC∞ (µg*h/mL)[§]	34400	3400	9.9
% AUC Extrapol	9.01	4.86	53.9
CL (mL/kg/h)	0.322	0.032	9.9
Vss (mL/kg)	79.9	12.5	15.7
Vz (mL/kg)	88.4	12.7	14.4
MRT (h)	251	59.9	23.8
C_0/Dose (µg/mL)	35.7	1.85	5.2
AUC_{last}/Dose (µg*h/mL)	2840	179	6.3
AUC∞/Dose (µg*h/mL)	3130	310	9.9
T 1/2.z (h)	193	44.2	22.9

C_0: back extrapolated to y-axis serum concentration after IV dosing, i.e. maximum concentration; AUC_{last}: Area under serum concentration vs. time curve up to the last detectable concentration; AUC∞: Area under serum concentration vs. time curve up to infinite time; AUC Extrapol: percentage of the area under the curve extrapolated to infinity; CL: Serum Clearance; Vss: Volume of distribution at steady-state; Vz: Volume of distribution on the terminal phase; MRT: Mean Residence Time; C_0/Dose: C_0 normalized to 1 mg/kg; AUC_{last}/Dose: Area under serum concentration vs. time curve up to the last detectable concentration normalized to 1 mg/kg; AUC∞/Dose: Area under serum concentration vs. time curve up to infinite time normalized to 1 mg/kg; $T_{1/2,z}$ Terminal half-life.

[§]Interval: 0-672 hrs.
